# Hydration of dicalcium silicate and diffusion through neo-formed calcium-silicate-hydrates at weathered surfaces control the long-term leaching behaviour of basic oxygen furnace (BOF) steelmaking slag

**DOI:** 10.1007/s11356-018-1260-7

**Published:** 2018-01-25

**Authors:** Douglas I. Stewart, Andrew W. Bray, Gideon Udoma, Andrew J. Hobson, William M. Mayes, Mike Rogerson, Ian T. Burke

**Affiliations:** 1 School of Civil Engineering, University of Leeds, Leeds, LS2 9JT UK; 2 School of Earth and Environment, University of Leeds, Leeds, LS2 9JT UK; 3 School of Environmental Sciences, University of Hull, Cottingham Road, Hull, HU6 7RX UK

**Keywords:** Steel slag, Vanadium, Alkaline waste, Leaching, Particle size, Reuse

## Abstract

Alkalinity generation and toxic trace metal (such as vanadium) leaching from basic oxygen furnace (BOF) steel slag particles must be properly understood and managed by pre-conditioning if beneficial reuse of slag is to be maximised. Water leaching under aerated conditions was investigated using fresh BOF slag at three different particle sizes (0.5–1.0, 2–5 and 10 × 10 × 20 mm blocks) and a 6-month pre-weathered block. There were several distinct leaching stages observed over time associated with different phases controlling the solution chemistry: (1) free-lime (CaO) dissolution (days 0–2); (2) dicalcium silicate (Ca_2_SiO_4_) dissolution (days 2–14) and (3) Ca–Si–H and CaCO_3_ formation and subsequent dissolution (days 14–73). Experiments with the smallest size fraction resulted in the highest Ca, Si and V concentrations, highlighting the role of surface area in controlling initial leaching. After ~2 weeks, the solution Ca/Si ratio (0.7–0.9) evolved to equal those found within a Ca–Si–H phase that replaced dicalcium silicate and free-lime phases in a 30- to 150-μm altered surface region. V release was a two-stage process; initially, V was released by dicalcium silicate dissolution, but V also isomorphically substituted for Si into the neo-formed Ca–Si–H in the alteration zone. Therefore, on longer timescales, the release of V to solution was primarily controlled by considerably slower Ca–Si–H dissolution rates, which decreased the rate of V release by an order of magnitude. Overall, the results indicate that the BOF slag leaching mechanism evolves from a situation initially dominated by rapid hydration and dissolution of primary dicalcium silicate/free-lime phases, to a slow diffusion limited process controlled by the solubility of secondary Ca–Si–H and CaCO_3_ phases that replace and cover more reactive primary slag phases at particle surfaces.

**Electronic supplementary material:**

The online version of this article (10.1007/s11356-018-1260-7) contains supplementary material, which is available to authorized users.

## Introduction

Steelmaking slag is an important industrial by-product, with an annual global production of 170–250 million tonnes (van Oss 2016). It is produced when CaO (or limestone/dolomite) is added to the steel furnace as a flux that reacts with process impurities (primarily silica) and separates them from the molten steel (Bobicki et al. 2012; Eloneva et al. 2010). The slag produced is named after the steelmaking process in which it is generated: basic oxygen furnace (BOF), electric arc furnace (EAF) and ladle furnace (LF) (Bobicki et al. 2012; Bonenfant et al. 2008; De Windt et al. 2011; Navarro et al. 2010; Wang et al. 2010). At the start of the twenty-first century, more than 60% of the world’s steel is produced by the basic oxygen process, with the remaining production by EAF (Smil 2006).

BOF slag, which is sometimes called basic oxygen steelmaking or Linz-Donawitz slag, has a composition that varies slightly with the iron source and the processing details, but its bulk chemical composition is relatively consistent between locations worldwide. The major element chemistry is dominated by Ca, Fe and Si, with lesser amounts of Mg, Mn and Al (Hobson et al. 2017; Shen et al. 2009; Tossavainen et al. 2007; Yildirim and Prezzi 2011). The mineralogical composition can be complex but BOF slag typically contains Ca-rich silicates (e.g. larnite, β-Ca_2_SiO_4_; merwinite, Ca_3_Mg(SiO_4_)_2_; rankinite, Ca_3_Si_2_O_7_; akermanite, Ca_2_MgSi_2_O_7_), Ca–Fe–A oxides (e.g. brownmillerite, Ca_2_FeAlO_5_; srebrodolskite, Ca_2_Fe_2_O_5_), periclase (MgO) and refractory Fe–Mg–Mn–Ca oxides (e.g. Wüstite, FeO) together with unused free lime (CaO) (Geiseler 1996; Hobson et al. 2017; Mayes et al. 2008; Piatak et al. 2015; Roadcap et al. 2005). EAF slag has a similar chemical composition to BOF slag, although its composition varies slightly with the type of scrap steel used, and it contains similar mineral phases (Tossavainen et al. 2007; Yildirim and Prezzi 2011).

The production of slag is an inevitable consequence of steel manufacturing, but this need not result in its disposal as waste, as steel slags have been used as secondary raw materials for more than 100 years (EuroSlag 2006). However, steel slag reuse rates vary enormously around the world (published reuse rates rarely differentiate between chemically similar BOF and EAF slags). For example, only 22% of steel slag is reused in China (Yi et al. 2012) and 30% is reused in India (Tiwari et al. 2016) whereas in Europe more than 75% of slag is reused (EuroSlag 2012), and in the USA 90% of steel slag is reused (van Oss 2016). These differences probably reflect differences in national policy and regulatory environments, which in the EU and USA promote the use of alternative raw materials; however, even in these markets, steel slag commands a low unit value (EuroSlag 2006; van Oss 2016).

The largest market for BOF slag is an aggregate for civil engineering applications, such as road construction (Ahmedzade and Sengoz 2009; Geiseler 1996; Huang et al. 2007; Qiang and Peiyu 2010; Yi et al. 2012). The most important properties of such aggregates are particle shape, strength, bulk density, volume stability, water absorption characteristics, resistance to breakdown during freeze/thaw cycles, crushing resistance and (for highway surface layers) resistance to abrasion and polishing (ASA 2002; Motz and Geiseler 2001). Many of these properties of slag-based aggregates are comparable with, or even better than, those of natural aggregates (Motz and Geiseler 2001), but concerns about the volume stability of BOF slag can prevent its immediate use in significant quantities (Sasaki and Hamazaki 2015). Free (unhydrated) lime (CaO) and periclase (MgO) in BOF slag can result in volumetric expansion upon hydration (Ahmedzade and Sengoz 2009; Motz and Geiseler 2001; Wang et al. 2010). Therefore, limits are sometimes placed on the free-lime content of BOF slag, or a period of slag conditioning is required before reuse is permitted (ASA 2002; Dippenaar 2004; Huang et al. 2007; Wang et al. 2010). Typically, it is stockpiled for a period of months, and rainfall (or other water) is allowed to infiltrate to promote the hydration process (Dippenaar 2004). In the UK, a construction company is processing and conditioning BOF slag produced at a major steelworks in the North of England to produce an aggregate for road construction (UKEA 2014a, b). Here de-metallised BOF slag is crushed and screened to < 20 mm and left to weather in windrows for > 6 months, to reduce the free-lime content and make it suitable for road construction (UKEA 2014a, b).

When BOF slag comes into contact with water (e.g. when rainwater infiltrates a slag stockpile), it produces highly alkaline leachate. Two processes generate alkalinity: rapid hydration and subsequent dissociation of free lime, and slower dissolution of periclase (if present) and Ca-silicates minerals, such as rankinite, larnite and akermanite (Mayes et al. 2008; Roadcap et al. 2005). Steel slags can contain trace metals from the primary ore, such as Al, Cr, Mo and V, which become concentrated in the slag by processing (steel slag production is about 10 to 15% of steel output; van Oss 2016). The fate of these trace metals during slag leaching depends both on the chemical form of the trace metal and on the stability of the specific host phases present in the slag. For example, vanadium can be hosted by dicalcium silicate phases as V(V) in tetrahedrally coordinated silicate sites, and by dicalcium aluminoferrite phases as both V(III) and V(IV) in octahedrally coordinated Fe(III) sites and as V(V) in tetrahedrally coordinated silicate sites (Chaurand et al. 2007a, b; Hobson et al. 2017). Dissolution of dicalcium silicates can therefore release V(V) to solution to produce aqueous orthovanadate species at high pH (Wehrli and Stumm 1989), which may subsequently be precipitated in neo-formed phases (oxyanions CrO_4_^2−^ and AsO_4_^3−^ with a similar tetrahedral structure can substitute for silicate in calcium silicate hydrates; Cornelis et al. 2008) or be released from the slag. Uncontrolled leaching of steel slag at abandoned sites has resulted in water containing up to 100 μg L^−1^ of vanadium entering local water courses (33 ± 25 μg L^−1^, *n* = 12; Mayes et al. 2008; Riley and Mayes 2015; Roadcap et al. 2005).

Currently, there is significant uncertainty about the factors controlling the kinetics of slag leaching, particularly for aggregate-sized slag particles, where the formation of surface alteration layers on the slag significantly affects the leaching process (Costa et al. 2016; Hobson et al. 2017; Huijgen and Comans 2006; Huijgen et al. 2005; Nikolić et al. 2016). This makes it difficult to specify the optimum weathering/leaching regime for slag conditioning (for example the recommended weathering period for BOF slag varies widely between 1 month and 12 months; ASA 2002; Dippenaar 2004; Huang et al. 2007; UKEA 2014a; Wang et al. 2010). Also, the release of potentially toxic trace elements, such as Cr and V, from slag is not widely perceived as an issue, provided release occurs primarily during slag conditioning where the generated leachate can be managed. However, this implies that better understanding is needed of how trace metal release evolves over time with the geochemistry of the system to ensure that trace metals do not become an issue that adversely affects the beneficial reuse of slag.

This study will investigate leachate generation processes in BOF slag–water–air systems as a function of time (up to 3 months) and assess the effect of changing particle size on leachate generation. In addition, slag mineral alteration and the development of an alteration rind will be investigated using electron microscopy. The effect of pre-treatment (6 months total immersion in aerated water; a well-defined weathering period representative of 12- to 18-months of rainfall infiltration) will be determined with respect to the potential for further leachate generation after the initial treatment process.

## Methods and materials

### Sample collection and preparation

BOF steel slag was collected in May 2013, within 1 week of its deposition, from the Yarborough Landfill at the Tata Steel Europe steelworks in Scunthorpe, UK (lat. 53° 35′ 22.24″ long. 0° 35′ 41.52″). The initial sample consisted of irregularly shaped 50- to 500-g blocks. Three 10 × 10 × 20 mm cuboids of BOF slag were cut from intact slag blocks using a diamond saw. Two were used directly in the leaching tests reported below (which will be referred to as the blocks), whereas one was soaked in deionised water (DIW; 18 MΩ; 2 L; Hobson et al. 2017) that was continually aerated using an aquarium pump in a glass Duran bottle stoppered with an air-permeable foam bung for 6 months prior to use (the pre-weathered block). Water losses due to evaporation were regularly replaced by deionised water. The remainder of the BOF steel slag sample was crushed using a jaw crusher and sieved into separate size fractions. Two size fractions were retained for testing: 0.5–1 mm (sand particles) and 2–5 mm (fine gravel particles). This provided three sized fractions representative of medium and fine gravels and sand used in standard aggregate mixtures for highway base and subbase layers (ASTM 2001).

### Leaching tests

Triplicate crushed slag samples (~2 g) were placed in 250 ml Duran bottles (Duran Group) containing DIW (~200 mL) to produce a liquid to solid ratio of 10 g L^−1^. The three 10 × 10 × 20 mm blocks (one pre-weathered, each ~8 g) were placed in 1-L Duran bottles containing DIW (~800 mL) to produce the same liquid to solid ratio. The Duran bottles were stoppered with air-permeable foam bungs to allow interaction with the atmosphere. The Duran bottles were kept on the lab bench at roughly 20 °C. Periodically (after 0, 1, 2, 5, 8, 14, 28, 57 and 73 days), the bottles were gently swirled, allowed to settle until the supernatant was visually clear and then measured for pH and conductivity. Solution samples (1 mL) were taken of the clear supernatant from each replicate and acidified with 5% HNO_3_ (9 mL; AnalaR NORMAPUR, VWR) before ICP-OES analysis. After sampling, the experiments were made up to their initial volumes with DIW. After the final solution samples were collected, the entire solid fractions were recovered using a 90-mm Buckner funnel and micro-glass fibre filters (Fisherbrand MF200-90).

### Scanning electron microscopy

The recovered slag fractions were set in Araldite® epoxy resin (Huntsman Advanced Materials), and the surface of the resulting resin blocks were polished using 3-, 1-, and 1/4-μm diamond paste (Struers) to expose the slag pieces in cross-section. Back-scatter electron micrographs were collected on a Quanta FEG 650 scanning electron microscope (SEM), which was equipped with an Oxford Instruments INCA 350 energy-dispersive X-ray spectroscopy (EDS) system controlled by AZtec acquisition software (i.e. for semi quantitative elemental analysis and mapping applications; elemental composition data was calibrated using a Co metal target). The AZTec software only reports EDS peaks that are > 3σ above baseline noise and uses theoretical element peak area relationships to deconvolute any overlapping EDS peaks (e.g. to avoid overestimation of the V Kα peak area in the presence of an overlapping Ti Kβ peak). Calibrated EDS data from each spot analysis was converted to mol%, and the total elemental abundance was normalised to 100% to allow determination of stoichiometric ratios (e.g. Ca/Si, V/Si etc.) within the phases analysed. Limits of detection are element specific, but normally were between 0.1–0.2 mol%. The average surface alteration depth for each sample was determined using perpendicular measurements at 50-μm intervals along the surface seen in 8–10 separate SEM images from each sample (*n* = 80–120; SI Fig. S1).

### Geochemical analysis

Solution pH and conductivity were measured using a Hach HQ40d multi-parameter meter and regularly calibrated electrodes. Elemental concentrations in aqueous solutions were determined by inductively coupled plasma atomic emission spectroscopy using a Thermo Fisher iCAP 7400 Radial ICP-OES (limit of detection for each element is presented in SI Table S1). Major and minor element composition of solid samples was determined by x-ray fluorescence (XRF) spectroscopy using an Olympus X-5000 XRF analyser. Mineralogical analysis was performed using a Bruker D8 X-ray diffractometer (XRD) using Cu K-alpha radiation.

## Results

### Slag composition

The elemental composition of the slag was dominated by Ca, Fe and Si with Mn and Mg as minor constituents (Table 1). XRD analysis (SI Fig. S2) showed it contains phases structurally matched to larnite (dicalcium silicate; β-Ca_2_SiO_4_), brownmillerite (dicalcium aluminoferrite; Ca_2_(Al, Fe)_2_O_5_), free-lime (CaO) and a Wüstite-like phase (FeO).

**Table 1:** Major element composition of freshly deposited Yarborough BOF steel slag (21 samples) determined by XRF

Nominal oxide	% *w*/*w*
CaO	40 ± 5.4
FeO	32 ± 9.4
SiO_2_	14 ± 3.4
MgO	5.2 ± 1.1
MnO	4.5 ± 0.8
Al_2_O_3_	1.2 ± 0.4
P_2_O_5_	1.3 ± 0.4
V_2_O_5_	0.81 ± 0.24
TiO_2_	0.30 ± 0.13
Cr_2_O_3_	0.24 ± 0.13
SO_3_	0.23 ± 0.09
Total	98.7

### Leaching tests

**Fig. 1:**
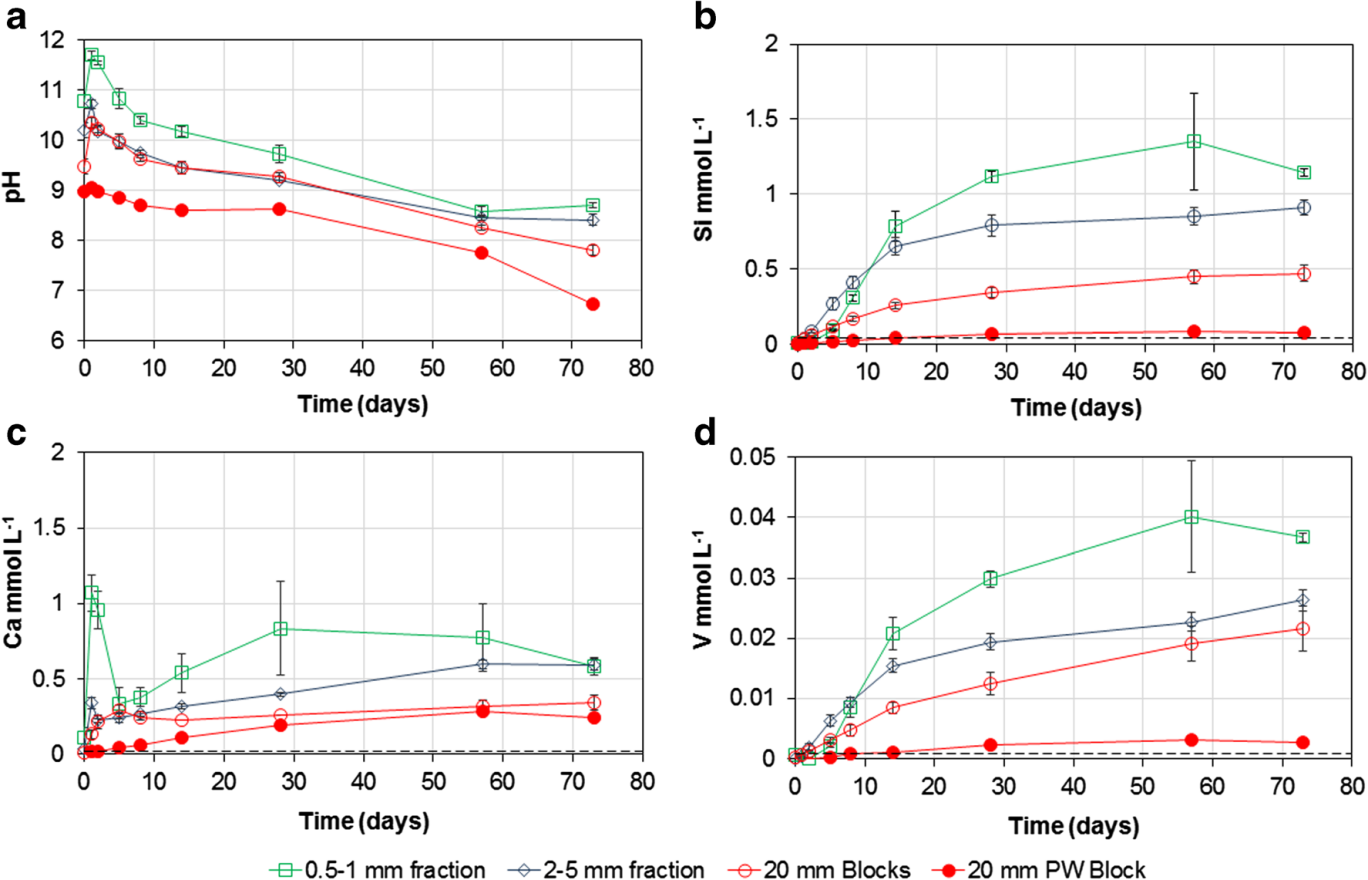
Evolution of solution pH, and aqueous Ca, Si and V concentrations (mmol L^−1^) measured in leaching tests using three different sized BOF slag particles. Dashed lines show the limit of detections. Error bars show 1 standard deviation of triplicate measurements; where not shown, error bars are less than the size of the symbols used

The solution pH behaved similarly in the leaching tests of all the BOF slag size fractions (Fig. 1). After 20 min of reaction, the pH of all tests was ≥ 9 (including the weathered block test), the maximum pH value was recorded after ~1 day and then the pH value gradually decreased with time. The sand-sized slag fraction reached the highest pH value after 24 h (pH 11.7), and the gravel-sized fraction and the blocks reached progressively lower maximum values (10.7 and 10.4 respectively). The pre-weathered block rapidly buffered the solution to pH 9.0, but only a very modest further increase over the first 24 h (to pH 9.1) before the pH decreased steadily until the end of the test.

The concentration of calcium ([Ca] mmol L^−1^) in the leaching tests on fresh slag exhibited a similar pattern with time for all the size fractions (Fig. 1(c)). Initially, there was a rapid increase in [Ca] to a short-term peak and a decrease in [Ca] at intermediate times, followed by a subsequent increase in [Ca] in the long term. Generally, the [Ca] was highest in the leaching test on the sand-sized fraction, and progressively lower with increasing slag particle size. The peak [Ca] in the leaching test on the sand-sized fraction occurred after 24 h (1.1 mmol L^−1^), and, unlike the tests on other size fractions, was larger than in the long-term [Ca] (0.58 mmol L^−1^). With the gravel-sized fraction, the short-term peak also occurred after 24 h (0.34 mmol L^−1^), but was less pronounced and exceeded by the long-term [Ca] (0.60 mmol L^−1^). With the block samples, the short-term peak (0.30 mmol L^−1^) occurred after 5 days, was quite modest in magnitude and was just exceeded by the long-term [Ca] (0.34 mmol L^−1^). The leaching test on the pre-weathered block exhibited no short-term peak, just a gradual increase in the [Ca] with time, reaching a final [Ca] just slightly lower than other blocks (0.25 mmol L^−1^).

The variation in silicon concentrations ([Si], mmol L^−1^) with time exhibited a similar pattern in all the leaching tests (Fig. 1(b)), a gradual increase in concentration with time to a near constant value after 73 days. Typically, at any time point, the [Si] was largest with the smallest size fraction (sand-sized), decreased with increasing particle size and was smallest with the pre-weathered block (the final concentrations were 1.1, 0.91, 0.47 and 0.08 mmol L^−1^ for sand-sized, fine gravel-sized, blocks and pre-weathered block, respectively). Aqueous [Ca]/[Si] ratios were consistently > 1 during the early part of the tests (< 5 days) for each size fraction (SI Fig. S3). However, at later time points (> 14 days), the [Ca]/[Si] ratios are consistently between 0.7 and 0.9 (Table 2, SI Fig. S3) for all size fractions except the pre-weathered block ([Ca]/[Si] > 1 for the entire test).

**Table 2:** SEM-EDS element ratios determined in the unreacted Ca_2_SiO_4_ phases and the Ca–Si–H phase found in slag particle surface layers after 73 days water leaching, and corresponding aqueous solution ratios calculated for solutions in contact with the slag particles

Fraction	Phase	Ca/Si^#^	V/Si^#^	*n*
Sand fraction 0.5–1 mm	Ca_2_SiO_4_	2.31 ± 0.02	0.011 ± 0.004	7
	Bulk Ca–Si–H	0.72 ± 0.29	0.008 ± 0.005	37
	Ca–Si–H Surface layer*	0.71 ± 0.15	0.006 ± 0.002	7
	Aqueous solution^	0.70 ± 0.23	0.033 ± 0.003	12
Gravel fraction 2–5 mm	Ca_2_SiO_4_	2.24 ± 0.02	0.010 ± 0.002	6
	Bulk Ca–Si–H	1.12 ± 0.54	0.009 ± 0.005	31
	Ca–Si–H surface layer*	0.73 ± 0.30	0.006 ± 0.003	4
	Aqueous solution^	0.67 ± 0.11	0.029 ± 0.002	12
Blocks 20 × 10 × 10 mm	Ca_2_SiO_4_	2.30 ± 0.16	0.029 ± 0.021	17
	Bulk Ca–Si–H	1.40 ± 0.32	0.051 ± 0.033	21
	Ca–Si–H surface layer*	1.15 ± 0.22	0.021 ± 0.012	4
	Aqueous solution^	0.89 ± 0.10	0.045 ± 0.006	8

^#^Element ratio uncertainty is 1 SD

*0–3 μm from particle surface

^average of data after 14 days reaction

*n* is the number of EDS measurements for each phase

The vanadium concentration ([V], mmol L^−1^) during leaching tests (Fig. 1d) exhibits very similar patterns to the [Si] concentrations, except [V] concentrations are approximately 4% of [Si] for each size fraction ([V]/[Si] ratios varied from 0.03 to 0.05 throughout each test; Table 1, SI Fig. S3). As with [Si], the [V] was highest in the sand-sized fraction tests. The concentrations of Na, Mg, K, Fe, Al, P, Cr, Mn, Ti, Zn and As were also measured and largely below detection limit in all leaching tests (SI Table S2).

### SEM analysis of slag recovered from the leaching tests

**Fig. 2:**
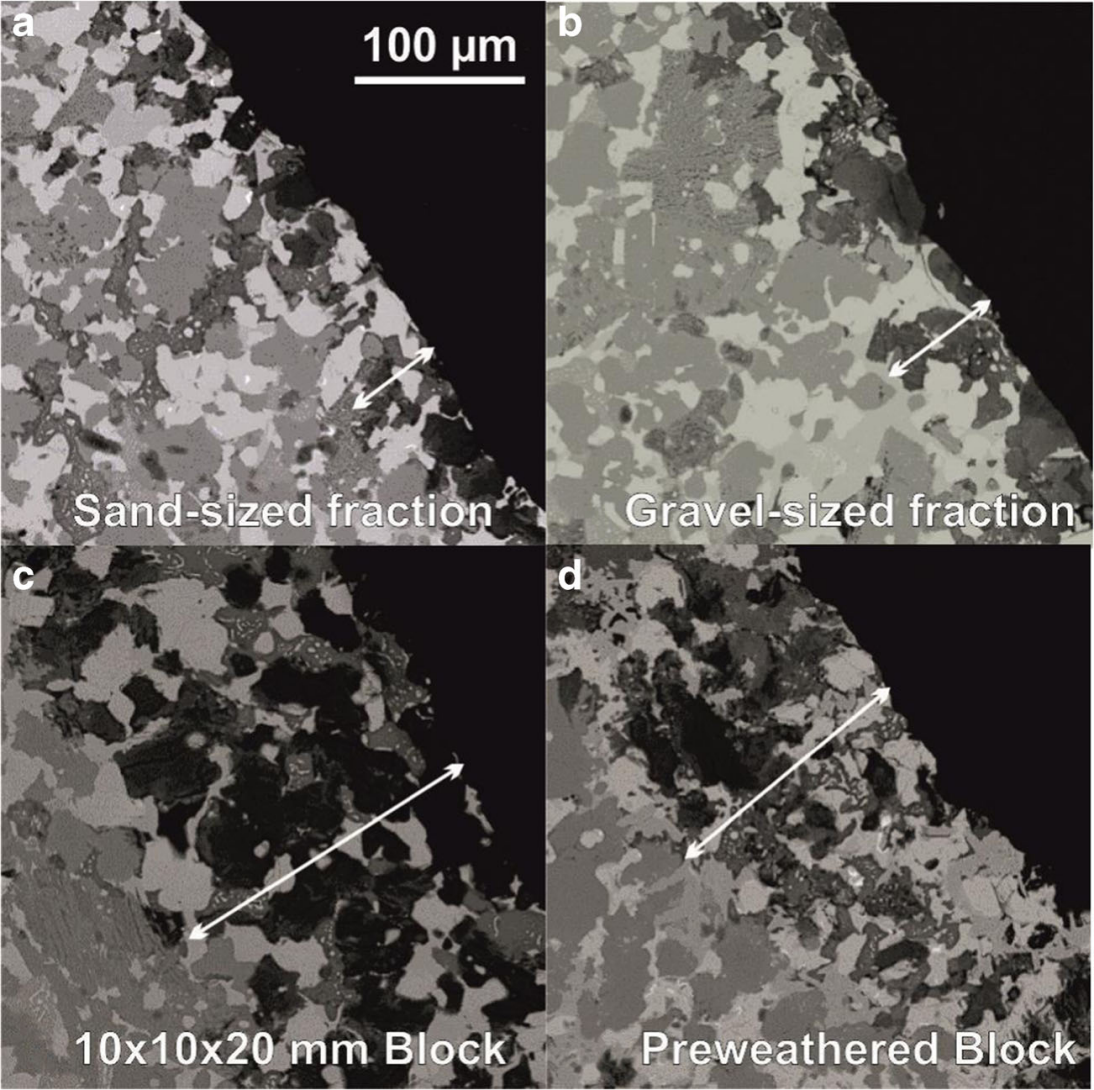
Example backscattered electron micrographs collected from the alteration zones present in different sized BOF slag particles after leaching for 73 days. All images shown at the same scale, white arrows indicate measured alteration thickness

SEM analysis showed that the centre of slag particles consists an interlocking crystalline matrix of typically 10- to 50-μm grains (Fig. 2; SI Fig. S1). The major phases identified by XRD analysis were also confirmed by EDS spot analysis (SI Fig. S4). The free-lime phase was substituted with Fe, Mn, Mg and Sc; the Wüstite-like phase contained Fe, Mn, Mg and Ca; the dicalcium silicate phase also contained a range of trace elements including P, Fe and V (SI Table S3), and the dicalcium aluminoferrite phase also contained Ti, Mn, Cr and V.

All size fractions exhibited an alteration zone around the edge of particles, visible on back-scatter electron micrographs (Fig. 2). The average alteration zone depth (as delineated by the unaltered dicalcium aluminoferrite and refractory oxide fragments) was highly variable within slag fractions, but generally increased with increasing particle size from ~30 μm on the sand-sized particles to ~150 μm on the blocks (Table 3). The alteration zone depth on the surface of the pre-weathered block (~80 μm) was thinner (but within error) of the blocks despite the additional period of leaching. EDS spot analysis indicated that dicalcium aluminoferrite and refractory oxide phases remained in the altered zone, but free lime and dicalcium silicate are largely absent, replaced by a low-density Ca- and Si-containing phase, previously identified as a calcium-silicate-hydrate phase (Ca–Si–H; SI Table S3; Hobson et al. 2017). SEM images of the block samples (SI Fig. S5a) indicated that there were occasional voids within the specimens remote from the block surface, and that some of these voids contained Ca–Si–H after weathering. Furthermore, an additional Ca- and O-containing layer (presumed to be CaCO_3_) was occasionally seen on the surface of selected sand-sized slag particles (SI Fig. S5b), and the pre-weathered block (SI Fig. S1 and S4).

**Table 3:** Thickness of the weathered zone on different sized BOF slag particles after leaching for 73 days

Slag fraction	Size range	Specific surface area (m^2^/kg)	Alteration depth (μm)^#^	*n*
Sand	0.5–1 mm	1.7–3.3*	31 ± 20	111
Gravel	2–5 mm	0.3–0.7*	57 ± 22	90
Block	10 × 10 × 20 mm	0.15	147 ± 74	115
Pre-weathered block	10 × 10 × 20	0.15	80 ± 34	80

^#^Alteration depth uncertainty is 1 SD

*Assumes cuboid particles and a slag density of 3600 kg/m^3^

*n* is the number of alteration depth measurements

**Fig. 3:**
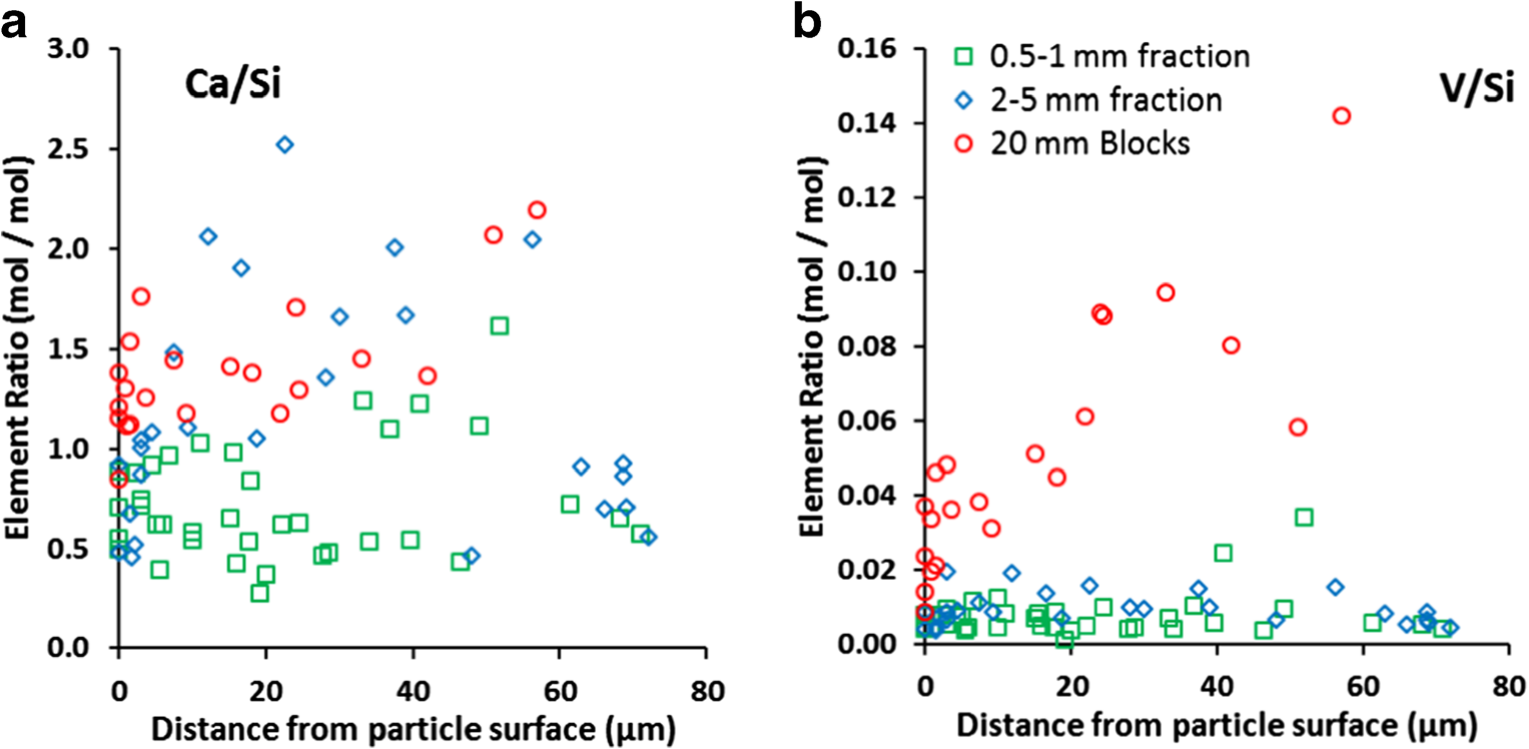
Distribution of SEM-EDS element ratios determined in the Ca–Si–H phase in the altered surface layer observed at the surface of slag particles after 73 days leaching

**Fig. 4:**
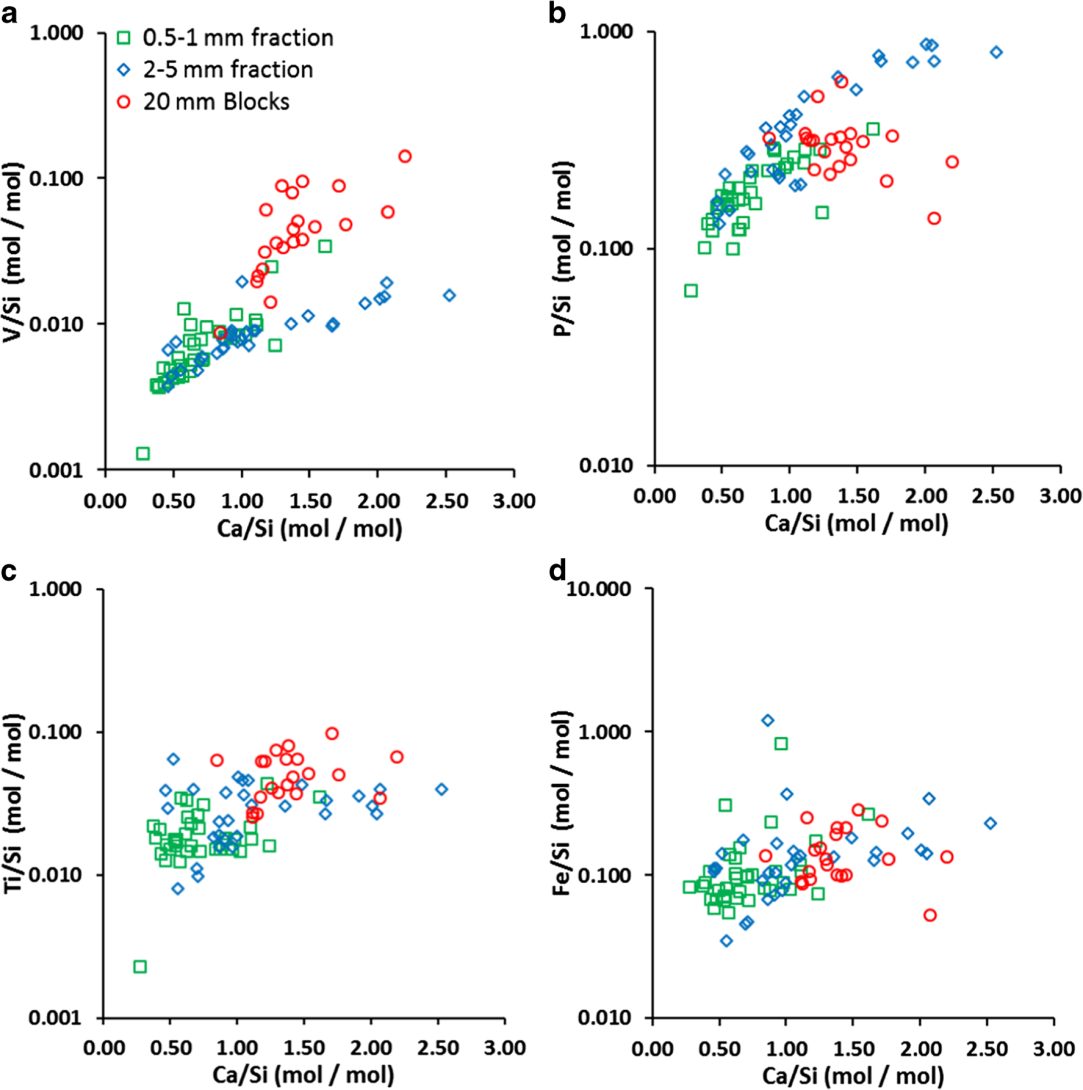
Variation in (A) V/Si, (B) P/Si, (C) Ti/Si and (D) Fe/Si as a function of Ca/SI ratio within the Ca–Si–H phases found in the surface alteration zone on slag particles

Detailed analysis of the Ca–Si–H layer present in the altered surface region of the slag particles showed considerable variation between samples (Fig. 3a). Ca/Si ratios were generally lower in the Ca–Si–H than that recorded in the dicalcium silicate phase (2.3 ± 0.2) and averaged 0.7, 1.1 and 1.4 respectively in the altered surface region found in the sand, gravel and blocks respectively (Table 2; SI Table S4). The Ca–Si–H phase was generally found to have an amorphous structure, except for parts of the alteration zone of the block samples, which had a blade-like morphology similar to the Ca–Si–H(II) phase jennite (Allen et al. 2007; Richardson 1999). The surface (0–3 μm) of the Ca–Si–H phase was Ca depleted relative to the bulk phase, except in sand-sized particles. V/Si ratios in the Ca–Si–H phases were generally within measurement error of the values found in the dicalcium silicates phase, although there was a slight trend to decreasing V/Si with decreasing Ca/Si ratio in the Ca–Si–H phase (Fig. 4(a)). This trend was also evident with some other trace elements within the Ca–Si–H (e.g. V, P, W, Al, Sc, Mg; see Fig. 4 for selected examples), which indicates preferential trace element uptake to Ca–Si–H with increasing Ca/Si ratio. However, other trace constituents (e.g. Fe, Mn, Ti) do not exhibit a strong trend with changes in Ca/Si of the Ca–Si–H phase.

## Discussion

### Surface alteration during BOF slag weathering under aerated conditions

Dicalcium silicate and free-lime dissolution constitute the initial sources of dissolved alkalinity, Ca and Si (and associated trace metals), during BOF slag weathering. The very high aqueous Ca/Si ratios observed in the first 5 days of the leaching tests (SI Fig. S3) most likely relate to a short period dominated by very rapid hydration and dissolution of free lime (Eq. 1 and 2; Shi et al. 2002).

1 $$ \mathrm{Ca}\mathrm{O}\ \left(\mathrm{s}\right)+{\mathrm{H}}_2\mathrm{O}\to \mathrm{Ca}{\left(\mathrm{OH}\right)}_2\ \left(\mathrm{s}\right) $$

2 $$ \mathrm{Ca}{\left(\mathrm{OH}\right)}_2\ \left(\mathrm{s}\right)\rightleftharpoons {\mathrm{Ca}}^{2+}\left(\mathrm{aq}\right)+{2\mathrm{OH}}^{-} $$

This is followed by a longer period (5–14 days) when [Si] and [Ca] were increasing and slower dicalcium silicate dissolution dominates (Eq. 3a; Taylor 1986);

3a $$ {\mathrm{Ca}}_2{\mathrm{SiO}}_4\left(\mathrm{s}\right)+{2\mathrm{H}}_2\mathrm{O}\to {2\mathrm{Ca}}^{2+}\left(\mathrm{aq}\right)+{\mathrm{H}}_2{{\mathrm{SiO}}_4}^{2-}\left(\mathrm{aq}\right)+{2\mathrm{O}\mathrm{H}}^{-} $$

pH <9

3b $$ {6\mathrm{Ca}}_2{\mathrm{Si}\mathrm{O}}_4\left(\mathrm{s}\right)+12{\mathrm{H}}_2\mathrm{O}\to {\mathrm{Ca}}_5{\mathrm{Si}}_6{\mathrm{O}}_{16}{\left(\mathrm{OH}\right)}_2\cdotp {4\mathrm{H}}_2\mathrm{O}\ \left(\mathrm{s}\right)+{7\mathrm{Ca}}^{2+}\ \left(\mathrm{aq}\right)+14{\mathrm{O}\mathrm{H}}^{-} $$

pH <11

3c $$ {6\mathrm{Ca}}_2{\mathrm{Si}\mathrm{O}}_4\left(\mathrm{s}\right)+14{\mathrm{H}}_2\mathrm{O}\to {\mathrm{Ca}}_9{\mathrm{Si}}_6{\mathrm{O}}_{18}{\left(\mathrm{OH}\right)}_6\cdotp {8\mathrm{H}}_2\mathrm{O}\ \left(\mathrm{s}\right)+3\mathrm{Ca}{\left(\mathrm{OH}\right)}_2\ \left(\mathrm{s}\right) $$

pH >12

(reactions 3b and c are simplified equations for the formation of tobermorite, Ca–Si–H(I) and jennite, Ca–Si–H(II), respectively).

Weathering of BOF slag produced an altered zone at the surface on the slag particles where free lime and dicalcium silicate were largely absent. In this zone, a new, low-density, Ca–Si–H phase formed between the unaltered fragments of dicalcium aluminoferrite and Wüstite. Ca–Si–H gel phases are the main products when dicalcium silicate reacts with water (Chen et al. 2004; Jennings 1986; Taylor 1986) and rapidly form from solution by heterogeneous nucleation (Garrault and Nonat 2001). In addition, the dicalcium silicate hydration mechanism is highly dependent on water availability, and therefore, there is propensity for oversaturation and retention of Ca(OH)_2_ within the alteration products (Eqs. 3b, 3c). There was no evidence for extensive Ca–Si–H phases forming beyond the original surface of the slag particles. There were occasional observations of alteration products in pore space within the slag (BOF slag has effective porosity of ~6% and accumulation of alteration products within voids is thought to contribute to the increased aggregate strength observed in post-weathered BOF slags; Wu et al. 2007).

An additional Ca-containing layer was occasionally observed on the surface of the slag particles that is consistent with calcium carbonate (CaCO_3_) which readily forms where alkaline Ca^2+^-rich solutions are in contact with atmospheric CO_2_ (Eq. 4).

4 $$ {\mathrm{Ca}}^{2+}\left(\mathrm{aq}\right)+{\mathrm{CO}}_2\left(\mathrm{aq}\right)+2{\mathrm{OH}}^{-}\leftrightarrows {\mathrm{Ca}\mathrm{CO}}_3\left(\mathrm{s}\right)+{\mathrm{H}}_2\mathrm{O} $$

At high pH, CaCO_3_ precipitation from Ca(OH)_2_ solutions is rapid and limited only by the rate of CO_2_ in-gassing and diffusion (Hodkin et al. 2016; Inskeep and Bloom 1985; Noyes et al. 1996). Thus, CO_2_ in-gassing and CaCO_3_ formation was responsible for the rapid reduction in [Ca] immediately following the period of free-lime dissolution and the general trend of reducing pH with time during these experiments.

### Effect of particle size on dicalcium silicate reaction

There was a clear difference in the Ca/Si ratio of the neo-formed Ca–Si–H phase in the slag leaching tests using sand, fine gravel, and the 10 × 10 × 20 mm blocks (0.7, 1.1 and 1.4 respectively). The Ca/Si ratio retained in Ca–Si–H phases gives a good indication of the solution chemistry in contact with the Ca–Si–H during precipitation. Ca–Si–H phases with a Ca/Si ratio < 1.0 form at relatively low pH (< 11), low [Ca] (< 2 mmol L^−1^) and high [Si] (> 1 mmol L^−1^) (Walker et al. 2016 reviewed the experimental conditions in 777 separate Ca–Si–H crystallisation experiments which produced phases with Ca/Si ratios ranging from 0.0 to 3.0). Therefore, the Ca–Si–H phase formed in the experiments using sand-sized particles is broadly consistent with the bulk solution chemistry measured after 5 days (Eq. 3b). The solution chemistry in the experiments using gravel particles and blocks also favour formation of Ca–Si–H with low Ca/Si ratios. However, the Ca/Si ratios in the altered zone of the fine gravel particles and medium gravel blocks were 1.1 and 1.4 respectively. Ca–Si–H phases with Ca/Si ratios > 1 indicate formation from solutions with progressively higher [Ca], lower [Si] and high pH (up to 20 mmol L^−1^ Ca, pH 12.5, and < 0.01 mmol L^−1^ Si; Walker et al. 2016). Therefore, the local microenvironment within the gel phase during Ca–Si–H precipitation must have been at considerable disequilibria with respect to the bulk solution in these experiments. As there was no obvious difference in slag composition between the experiments, the variation in the Ca–Si–H Ca/Si ratios must relate to altered surface region thickness.

After the leaching tests, the depth of alteration zone increased with decreasing slag surface area to volume ratio, i.e. alteration zone depth was greatest in the largest particles. This was probably because systems with larger slag surface area initially caused more rapid changes in the solution chemistry (resulting in higher [Ca] and [Si] in solution). Thus, when averaged over the test, the degree of disequilibria between the bulk solution and the alteration zone was lower in tests with larger surface area, resulting in slower progression of the alteration zone front into the smaller particles. For these particles, the shorter ion diffusion pathways led to formation of a Ca–Si–H phase in conditions close to equilibrium with the bulk solution. Conversely, for large particles, the thicker alteration zone indicates more rapid progression of the alteration front, which led to longer ion diffusion pathways and more difference between the chemistry of the alteration front and the bulk solution, and precipitation of Ca–Si–H with higher Ca/Si ratios (Eq. 3c; explaining the occasional observed jennite-like structures and composition found in the block samples).

After 2 weeks in all experiments, the rate of change in solution chemistry reduced and the aqueous Ca/Si ratio evolved from that expected from initial free-lime and dicalcium silicate dissolution (> 2) to values much closer to those found in the Ca–Si–H layer (0.7–0.9). This suggests that the rate of slag leaching had slowed significantly over the period of testing, probably as a result of Ca–Si–H formation in the altered zone, and CaCO_3_ on the slag surface. Thus, leaching of fresh slag likely becomes a diffusion-controlled process, limited by the rate at which water can diffuse through the neo-formed precipitates to the zone within the slag where free lime and dicalcium silicate are available to hydrate.

There is also evidence that the surface of the Ca–Si–H layer that was originally in contact with solution is depleted in Ca relative to the bulk of the Ca–Si–H layer. This may represent an incipient secondary alteration front forming within the Ca–Si–H layer. In Portland cement evolution, early formed Ca–Si–H(II) with an imperfect jennite structure containing Ca(OH)_2_ and a Ca/Si of 1.5–2.2 (typically 2.0; Chen et al. 2004; Gard and Taylor 1976) commonly recrystallizes as pore fluid composition evolves to lower [Ca] and lower pH to form Ca–Si–H(I) with an imperfect tobermorite structure with a Ca/Si of 0.67–1.5 (typically ~0.8; Chen et al. 2004). In these experiments, however, it is more likely that this surface alteration simply represents the preferential loss of more soluble elements (i.e. Ca) from the Ca–Si–H phase prior to full dissolution. Trace element ratios (e.g. V/Si and P/Si) within the Ca–Si–H were also notably lower in areas with lower Ca/Si ratios (Fig. 4). Therefore, Ca–Si–H alteration potentially also offers a mechanism for trace element release to solution.

### Controls on vanadium release during slag weathering

**Fig. 5:**
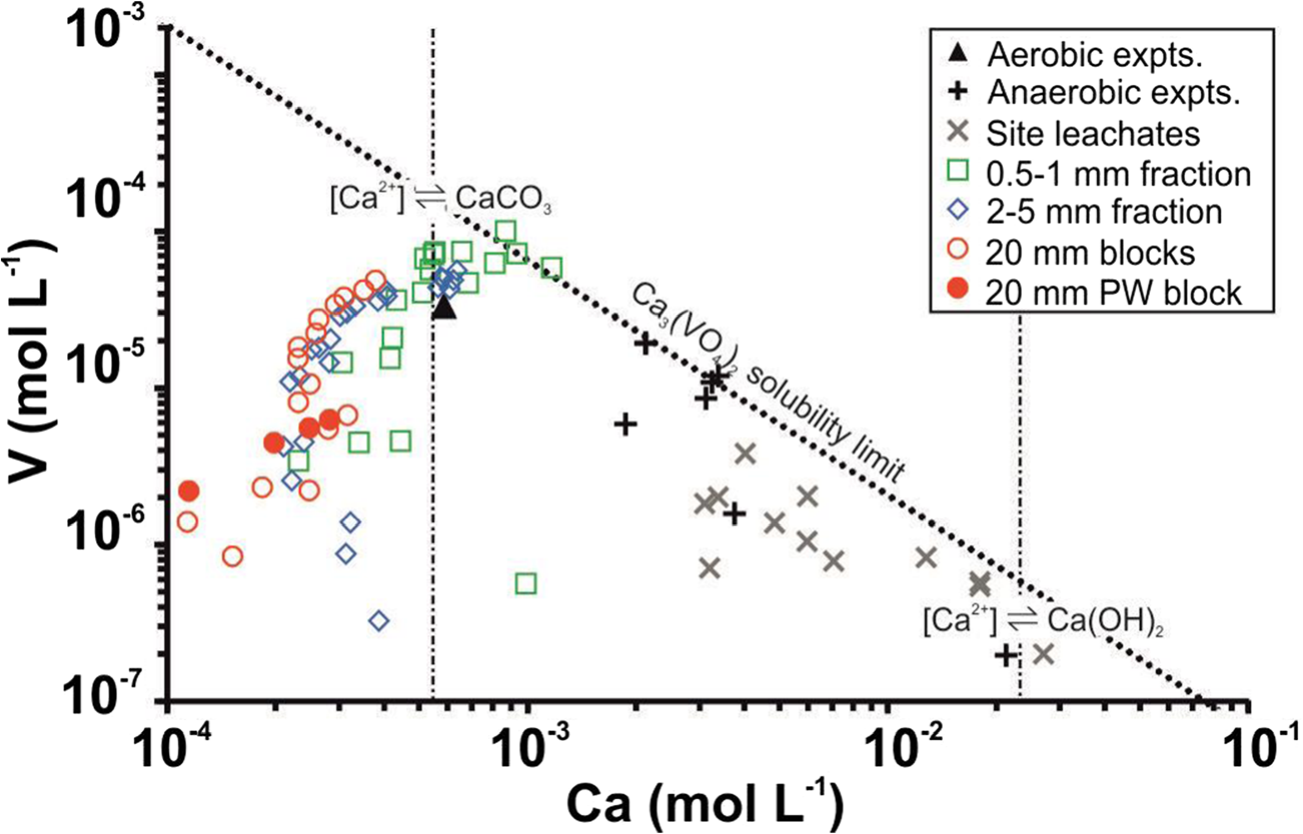
Plot of [V] versus [Ca] during the leaching tests on different BOF steel slag size fractions. Selected field data and end point data from other aerated and air-excluded leaching tests are shown for comparison (experimental data from De Windt et al. 2011; Hobson et al. 2017; Huijgen and Comans 2006; site data from Mayes et al. 2008; Riley and Mayes 2015; Roadcap et al. 2005). Dotted line marks the solubility limit for Ca_3_(VO_4_)_2_ at 20 °C (Log Ksp = − 17.97; Allison et al. 1991). Vertical dashed lines indicate [Ca] in solutions in equilibrium with calcite in contact with atmospheric CO_2_ or with Ca(OH)_2_ respectively (both at 20 °C)

V release from BOF slag is of concern due to its generally high concentration in slag (Chaurand et al. 2006; De Windt et al. 2011; Proctor et al. 2000; Tossavainen et al. 2007), its high aquatic toxicity as dissolved V(V) (Barceloux 1999; Jensen-Fontaine et al. 2014; Mišík et al. 2014) and the increased mobility of the vanadate oxyanion at high pH (Peacock and Sherman 2004; Wehrli and Stumm 1989). During BOF slag leaching, [V] is thought to be ultimately limited by Ca_3_(VO_4_)_2_ solubility (*K*_sp_ = 10^–17.97^; Allison et al. 1991; Cornelis et al. 2008; De Windt et al. 2011; Huijgen and Comans 2006; Schindler et al. 2000). Due to the inverse relationship of [Ca] and [V] implied by Ca_3_(VO_4_)_2_ solubility, and high [Ca] due to Ca(OH)_2_ equilibrium, [V] is normally limited to very low concentrations in slag leachates (Fig. 5). However, under aerated leaching conditions, [Ca] and hydroxide are removed by CaCO_3_ precipitation (Eq. 4), leading the conditions where V can accumulate in solution at much higher concentrations. In all the slag leaching experiments, the solution composition evolves with time towards equilibrium with both CaCO_3_ and Ca_3_(VO_4_)_2_. Experiments using sand-sized particles reach this end point most rapidly due to higher surface area, and total Ca_2_SiO_4_ dissolved, but the experiments with the gravel and block samples show significant progression towards that end point. The initial release of V to solution is due to dicalcium silicate dissolution (Eq. 3a) but as the reaction progresses, V is probably isomorphically substituted for Si within the neo-formed Ca–Si–H phase (Eq. 3b, 3c; as observed by Hobson et al. 2017). After 2 weeks, V release is reduced as slow Ca–Si–H dissolution becomes the dominant V leaching process in all experiments. This ultimate end point is most clearly illustrated by the leaching experiment using the pre-weathered block, in which the initial rapid dicalcium silicate/free lime-dominated leaching phase is not observed and solution chemistry is controlled by slower dissolution of secondary Ca–Si–H and CaCO_3_ phases present at the slag surface (with notably lower rates of V release).

### Implications of storage and reuse of BOF slag

There are three distinct phases during the weathering of BOF slag particles under aerated conditions. Firstly, a relatively short period when leaching is dominated by hydration and dissolution of free lime, secondly the hydration and dissolution of dicalcium silicate present at the surface of the slag particles and thirdly a longer period when leaching is primarily controlled by much slower dissolution of secondary phases that form on the surface of slag particles.

Although CO_2_ is available from atmosphere, the initial rate of leaching is such that high pH and high [Ca] leachates are rapidly produced, especially if fine high surface area particles are present within crushed slag gravels. Over time, CO_2_ in-gassing removes Ca and hydroxide, moderating solution pH and [Ca]. However, this produces conditions highly conducive to the accumulation of the V released from dicalcium silicate in solution (i.e. low [Ca] permits much higher [V] before equilibrium with Ca_3_(VO_4_)_2_ is reached). Therefore, it is important that during slag weathering (e.g. in windrows), the early formed leachates are carefully monitored and managed to avoid any potential for environmental harm. Potential environmental harm may be minimised by conducting initial BOF slag pre-conditioning under water-saturated conditions (limiting CO_2_ in-gassing, and limiting V accumulation in leachates), followed by leachate treatment (e.g. aeration) designed to promote CO_2_ in-gassing, Ca removal and pH reduction (Gomes et al. 2017).

After the initial weathering period, a Ca–Si–H surface layer was formed on the slag particles. The presence of this layer indicates a shift from rapid solubility controlled weathering of dicalcium silicate to a slower diffusion limited process. In addition, other slag phases embedded in the Ca–Si–H such as V-hosting brownmillerite (Chaurand et al. 2007a, b; Hobson et al. 2017), which becomes unstable below pH 8 (Engström et al. 2013), will be protected by buffering of micro-habitats within the surface region by Ca–Si–H to pH > 9, reducing the potential for metal leaching. Release of trace elements (including V) from the pre-conditioned blocks will largely depend on the dissolution/recrystallization rates of the Ca–Si–H host phases. However, the formation of thick alteration layers, and the fact that the Ca–Si–H is still present at the original slag surface after 9 months (in the pre-weathered block), indicates that Ca–Si–H weathering rates are slow. In addition, diffusion-limited hydration of dicalcium silicate may cause the Ca–Si–H layer to thicken over time, increasing the water diffusion pathway until dicalcium silicate hydration also becomes limited by Ca–Si–H dissolution rates. Thus, unless the BOF slag particles are physically broken to expose fresh surfaces, future generation of highly alkaline metal-rich leachates during after-use is not expected, leading to low potential for environmental harm during most after-use situations. However, an implication of the work is the need to protect slag particles from physical damage during reuse (e.g. by freeze thaw-cycling, etc.), as this could re-expose free-lime and dicalcium silicate phases to solution.

## Conclusions

Initial leaching of BOF slag under aerated conditions is dominated by rapid hydration and dissolution of the free-lime and dicalcium silicate phases present at particle surfaces. Preconditioning of BOF slag under fully aerated water promotes accumulation of V in solution. Therefore, initial leaching under air-excluded conditions is advisable as V concentrations are orders of magnitude lower in Ca(OH)_2_ saturated solutions (although this may necessitate separate leachate treatment to reduce alkalinity prior to leachate discharge). After a relatively short period (2 weeks under water immersion), solution chemistry becomes dominated by dissolution of the secondary Ca–Si–H and CaCO_3_ phases that replace and covered the primary slag phases at the surface of the particles. This isolates the more reactive primary slag phase in the interior of particles and slows the overall release of alkalinity and metals to solution, leading to much lower potential for environmental harm during after use in a range of engineering applications.

## Electronic supplementary material

XRD pattern from unreacted BOF slag, additional SEM images, EDS spectra and composition tables. Plots of aqueous elemental ratios in leaching tests. Collectively, the paper and these sources provide all the relevant data for this study.

ESM 1 (DOCX 5228 kb)
