# Supplementary material for: Hydration of dicalcium silicate and diffusion through neo-formed calcium-silicate-hydrates at weathered surfaces control the long-term leaching behaviour of basic oxygen furnace (BOF) steelmaking slag
Source: Environ Sci Pollut Res Int. 2018 Jan 25;25(10):9861–72. doi: 10.1007/s11356-018-1260-7 (PMC5891564; doi:10.1007/s11356-018-1260-7)
Supplement: Supplementary file 1 — (DOCX 5228 kb) [file 11356_2018_1260_MOESM1_ESM.docx]

**Supporting Information for:**

Hydration of Dicalcium Silicate and Diffusion through Neo-Formed Calcium-Silicate-Hydrates at Weathered Surfaces Control the Long-Term Leaching Behaviour of Basic Oxygen Furnace (BOF) Steelmaking Slag

Douglas I. Stewart^1*^, Andrew W. Bray^2^, Gideon Udoma^1^, Andrew J. Hobson^2^, William M. Mayes^3^, Mike Rogerson^3^ and Ian T. Burke^2^

^1^School of Civil Engineering, University of Leeds, LS2 9JT, UK

^2^School of Earth and Environment, University of Leeds, LS2 9JT, UK

^3^School of Environmental Sciences, University of Hull, Cottingham Road, Hull, HU6 7RX, UK

**Corresponding Author - E-mail: d.i.stewart@leeds.ac.uk; Phone: +44 113 3432287*

Prepared for submission to Environmental Science and Pollution Research

*Consists of 17 pages with 4 tables and 5 figures.*

**SI Table S1.** Limits of detection for each element measured by ICP-OES.

| Element | Limit of Detection |
| --- | --- |
|  | (mmol L^-1^) |
| Na | 0.2857 |
| Mg | 0.0178 |
| K | 0.0793 |
| Fe | 0.0075 |
| Si | 0.0416 |
| Al | 0.0312 |
| P | 0.0031 |
| V | 0.0008 |
| Cr | 0.0009 |
| Mn | 0.0011 |
| Ti | 0.0010 |
| Ca | 0.0204 |
| Zn | 0.0002 |
| As | 0.0004 |

**SI Table S2.** Experimental conditions and solution concentrations, as determined by ICP-OES. DL = below detection limit.

| Size Fraction | Day | pH | Conductivity | Na | Mg | K | Fe | Si | Al | P | V | Cr | Mn | Ti | Ca | Zn | As |
| --- | --- | --- | --- | --- | --- | --- | --- | --- | --- | --- | --- | --- | --- | --- | --- | --- | --- |
|  |  |  | (μS) | (mmol L^-1^) | | | | | | | | | | | | | |
| Sand (0.5-1.0 mm) | 0 | 10.9 | 53 | DL | DL | DL | 0.020 | DL | DL | DL | DL | DL | 0.004 | DL | 0.155 | DL | DL |
|  |  | - | - | - | - | - | - | - | - | - | - | - | - | - | - | - | - |
|  |  | 10.7 | 32 | DL | DL | DL | 0.008 | DL | DL | DL | DL | DL | 0.002 | DL | 0.064 | DL | DL |
|  | 1 | 11.8 | 790 | DL | DL | DL | DL | DL | DL | DL | DL | DL | DL | DL | 1.087 | DL | DL |
|  |  | 11.6 | 664 | DL | DL | DL | DL | DL | DL | DL | DL | DL | DL | DL | 0.944 | DL | DL |
|  |  | 11.7 | 725 | DL | DL | DL | DL | DL | DL | DL | DL | DL | DL | DL | 1.182 | DL | DL |
|  | 2 | 11.6 | 731 | DL | DL | DL | DL | DL | DL | DL | DL | DL | DL | DL | 1.064 | DL | DL |
|  |  | 11.5 | 575 | DL | DL | DL | DL | DL | DL | DL | DL | DL | DL | DL | 0.816 | DL | DL |
|  |  | 11.5 | 679 | DL | DL | DL | DL | DL | DL | DL | DL | DL | DL | DL | 0.987 | DL | DL |
|  | 5 | 11.0 | 186.1 | DL | DL | DL | DL | 0.091 | DL | DL | 0.002 | DL | 0.001 | DL | 0.443 | 0.001 | DL |
|  |  | 10.6 | 105.7 | DL | DL | DL | DL | 0.081 | DL | DL | 0.002 | DL | DL | DL | 0.231 | 0.004 | DL |
|  |  | 10.9 | 166.5 | DL | DL | DL | DL | 0.092 | DL | DL | 0.002 | DL | DL | DL | 0.340 | 0.001 | DL |
|  | 8 | 10.5 | 137.5 | DL | DL | DL | DL | 0.333 | DL | DL | 0.011 | DL | 0.001 | DL | 0.420 | DL | DL |
|  |  | 10.4 | 118.2 | DL | DL | DL | DL | 0.296 | DL | DL | 0.007 | DL | DL | DL | 0.306 | DL | DL |
|  |  | 10.4 | 119.7 | DL | DL | DL | DL | 0.292 | DL | DL | 0.008 | DL | DL | DL | 0.415 | DL | DL |
|  | 14 | 10.3 | 168.1 | DL | DL | DL | DL | 0.875 | DL | DL | 0.024 | DL | 0.003 | DL | 0.683 | DL | DL |
|  |  | 10.2 | 146.9 | DL | DL | DL | DL | 0.793 | DL | DL | 0.020 | DL | 0.001 | DL | 0.511 | DL | DL |
|  |  | 10.1 | 120.1 | DL | DL | DL | DL | 0.676 | DL | DL | 0.018 | DL | 0.002 | DL | 0.431 | DL | DL |
|  | 28 | 9.5 | 133.2 | DL | DL | DL | 0.028 | 1.161 | DL | DL | 0.031 | DL | 0.008 | DL | 0.810 | DL | DL |
|  |  | 9.8 | 127.5 | DL | DL | DL | DL | 1.100 | DL | DL | 0.029 | DL | DL | DL | 0.536 | DL | DL |
|  |  | 9.9 | 133 | DL | DL | DL | DL | 1.092 | DL | DL | 0.029 | DL | 0.001 | DL | 1.163 | DL | DL |
|  | 57 | 8.5 | 116.9 | DL | 0.019 | DL | 0.026 | 1.212 | DL | DL | 0.036 | DL | 0.006 | DL | 0.932 | DL | DL |
|  |  | 8.6 | 118.6 | DL | DL | DL | DL | 1.119 | DL | DL | 0.034 | DL | DL | DL | 0.518 | DL | DL |
|  |  | 8.7 | 122.9 | DL | DL | DL | 0.008 | 1.716 | DL | 0.004 | 0.051 | DL | 0.003 | DL | 0.867 | DL | DL |
|  | 73 | 8.8 | 119.9 | DL | DL | DL | DL | 1.155 | DL | DL | 0.037 | DL | DL | DL | 0.551 | DL | DL |
|  |  | 8.7 | 116.5 | DL | DL | DL | DL | 1.112 | DL | DL | 0.036 | DL | 0.001 | DL | 0.547 | DL | DL |
|  |  | 8.7 | 114.2 | DL | DL | DL | 0.012 | 1.165 | DL | DL | 0.037 | DL | 0.004 | DL | 0.655 | DL | DL |

**SI Table S2.** Continued

| Size Fraction | Day | pH | Conductivity | Na | Mg | K | Fe | Si | Al | P | V | Cr | Mn | Ti | Ca | Zn | As |
| --- | --- | --- | --- | --- | --- | --- | --- | --- | --- | --- | --- | --- | --- | --- | --- | --- | --- |
|  |  |  | (μS) | (mmol L^-1^) | | | | | | | | | | | | | |
| Gravel (2.0-5.0 mm) | 0 | 10.3 | 24.9 | DL | DL | DL | DL | DL | DL | DL | DL | DL | DL | DL | 0.038 | DL | DL |
|  |  | 10.2 | 20.59 | DL | DL | DL | DL | DL | DL | DL | DL | DL | DL | DL | 0.026 | DL | DL |
|  |  | 10.0 | 19.22 | DL | DL | DL | DL | DL | DL | DL | DL | DL | DL | DL | 0.034 | DL | DL |
|  | 1 | 10.8 | 156.9 | DL | DL | DL | DL | DL | DL | DL | DL | DL | DL | DL | 0.386 | DL | DL |
|  |  | 10.7 | 132 | DL | DL | DL | DL | DL | DL | DL | DL | DL | DL | DL | 0.322 | DL | DL |
|  |  | 10.7 | 134.4 | DL | DL | DL | DL | DL | DL | DL | DL | DL | DL | DL | 0.313 | DL | DL |
|  | 2 | 10.3 | 89.7 | DL | DL | DL | DL | 0.070 | DL | DL | 0.001 | DL | DL | DL | 0.221 | DL | DL |
|  |  | 10.2 | 94.1 | DL | DL | DL | DL | 0.083 | DL | DL | 0.002 | DL | DL | DL | 0.239 | DL | DL |
|  |  | 10.1 | 86.2 | DL | DL | DL | DL | 0.091 | DL | DL | 0.002 | DL | DL | DL | 0.210 | DL | DL |
|  | 5 | 10.0 | 85.9 | DL | DL | DL | DL | 0.255 | DL | DL | 0.006 | DL | DL | DL | 0.233 | DL | DL |
|  |  | 9.8 | 80.2 | DL | DL | DL | DL | 0.229 | DL | DL | 0.006 | DL | DL | DL | 0.219 | DL | DL |
|  |  | 10.1 | 96.4 | DL | DL | DL | DL | 0.315 | DL | DL | 0.007 | DL | DL | DL | 0.281 | 0.000 | DL |
|  | 8 | 9.8 | 91.1 | DL | DL | DL | DL | 0.398 | DL | DL | 0.009 | DL | DL | DL | 0.263 | DL | DL |
|  |  | 9.7 | 87 | DL | DL | DL | DL | 0.370 | DL | DL | 0.009 | DL | DL | DL | 0.251 | DL | DL |
|  |  | 9.8 | 92.7 | DL | DL | DL | DL | 0.456 | DL | DL | 0.010 | DL | DL | DL | 0.283 | DL | DL |
|  | 14 | 9.6 | 94.2 | DL | DL | DL | DL | 0.714 | DL | DL | 0.017 | DL | DL | DL | 0.332 | DL | DL |
|  |  | 9.4 | 88.5 | DL | DL | DL | DL | 0.594 | DL | DL | 0.014 | DL | DL | DL | 0.301 | DL | DL |
|  |  | 9.4 | 91.6 | DL | DL | DL | DL | 0.646 | DL | DL | 0.015 | DL | DL | DL | 0.314 | DL | DL |
|  | 28 | 9.2 | 144.7 | DL | DL | DL | DL | 0.859 | DL | DL | 0.021 | DL | DL | DL | 0.405 | DL | DL |
|  |  | 9.1 | 110.3 | DL | DL | DL | DL | 0.716 | DL | DL | 0.018 | DL | DL | DL | 0.384 | DL | DL |
|  |  | 9.3 | 110.9 | DL | DL | DL | DL | 0.801 | DL | DL | 0.019 | DL | DL | DL | 0.407 | DL | DL |
|  | 57 | 8.5 | 138 | DL | DL | DL | DL | 0.918 | DL | DL | 0.024 | DL | DL | DL | 0.620 | DL | DL |
|  |  | 8.4 | 131.5 | DL | DL | DL | DL | 0.824 | DL | DL | 0.022 | DL | DL | DL | 0.562 | DL | DL |
|  |  | 8.5 | 135.6 | DL | DL | DL | DL | 0.810 | DL | 0.003 | 0.022 | DL | DL | DL | 0.608 | DL | DL |
|  | 73 | 8.5 | 133.3 | DL | 0.019 | DL | DL | 0.967 | DL | DL | 0.028 | DL | DL | DL | 0.636 | DL | DL |
|  |  | 8.4 | 124.2 | DL | DL | DL | DL | 0.892 | DL | DL | 0.026 | DL | DL | DL | 0.572 | DL | DL |
|  |  | 8.3 | 128.2 | DL | DL | DL | DL | 0.876 | DL | DL | 0.025 | DL | DL | DL | 0.581 | DL | DL |

**SI Table S2.** Continued

| Size Fraction | Day | pH | Conductivity | Na | Mg | K | Fe | Si | Al | P | V | Cr | Mn | Ti | Ca | Zn | As |
| --- | --- | --- | --- | --- | --- | --- | --- | --- | --- | --- | --- | --- | --- | --- | --- | --- | --- |
|  |  |  | (μS) | (mmol L^-1^) | | | | | | | | | | | | | |
| Blocks (20 x 10 x 10 mm) | 0 | 9.6 | 11.89 | DL | DL | DL | DL | DL | DL | DL | DL | DL | DL | DL | DL | DL | DL |
|  |  | 9.4 | 11.57 | DL | DL | DL | DL | DL | DL | DL | DL | DL | DL | DL | DL | DL | DL |
|  | 1 | 10.5 | 84.3 | DL | DL | DL | DL | DL | DL | DL | DL | DL | DL | DL | 0.152 | DL | DL |
|  |  | 10.3 | 66.7 | DL | DL | DL | DL | DL | DL | DL | DL | DL | DL | DL | 0.114 | DL | DL |
|  | 2 | 10.3 | 100.3 | DL | DL | DL | DL | 0.059 | DL | DL | 0.001 | DL | DL | DL | 0.248 | DL | DL |
|  |  | 10.1 | 80.5 | DL | DL | DL | DL | 0.049 | DL | DL | 0.001 | DL | DL | DL | 0.183 | DL | DL |
|  | 5 | 10.1 | 106.5 | DL | DL | DL | DL | 0.124 | DL | DL | 0.003 | DL | DL | DL | 0.316 | DL | DL |
|  |  | 9.9 | 95.9 | DL | DL | DL | DL | 0.106 | DL | DL | 0.003 | DL | DL | DL | 0.279 | 0.001 | DL |
|  | 8 | 9.7 | 85.2 | DL | DL | DL | DL | 0.178 | DL | DL | 0.005 | DL | DL | DL | 0.249 | DL | DL |
|  |  | 9.6 | 81.5 | DL | DL | DL | DL | 0.153 | DL | DL | 0.004 | DL | DL | DL | 0.231 | DL | DL |
|  | 14 | 9.5 | 73.3 | DL | DL | DL | DL | 0.271 | DL | DL | 0.009 | DL | DL | DL | 0.231 | DL | DL |
|  |  | 9.4 | 72.8 | DL | DL | DL | DL | 0.240 | DL | DL | 0.008 | DL | DL | DL | 0.230 | DL | DL |
|  | 28 | 9.3 | 77.9 | DL | DL | DL | DL | 0.366 | DL | DL | 0.014 | DL | DL | DL | 0.263 | DL | DL |
|  |  | 9.2 | 79.2 | DL | DL | DL | DL | 0.321 | DL | DL | 0.011 | DL | DL | DL | 0.258 | DL | DL |
|  | 57 | 8.3 | 101 | DL | DL | DL | DL | 0.479 | DL | DL | 0.021 | DL | DL | DL | 0.351 | DL | DL |
|  |  | 8.2 | 94.2 | DL | DL | DL | DL | 0.415 | DL | DL | 0.017 | DL | DL | DL | 0.293 | DL | DL |
|  | 73 | 8.0 | 95.6 | DL | DL | DL | DL | 0.508 | DL | DL | 0.024 | DL | DL | DL | 0.379 | DL | DL |
|  |  | 7.6 | 87.3 | DL | DL | DL | DL | 0.434 | DL | DL | 0.019 | DL | DL | DL | 0.309 | DL | DL |
| Pre-weathered Block (20 x 10 x 10 mm) | 0 | 9.0 | 10.28 | DL | DL | DL | DL | DL | DL | DL | DL | DL | DL | DL | DL | DL | DL |
|  | 1 | 9.1 | 18.45 | DL | DL | DL | DL | DL | DL | DL | DL | DL | DL | DL | DL | DL | DL |
|  | 2 | 9.0 | 20.53 | DL | DL | DL | DL | DL | DL | DL | DL | DL | DL | DL | DL | DL | DL |
|  | 5 | 8.9 | 28.5 | DL | DL | DL | DL | DL | DL | DL | DL | DL | DL | DL | 0.042 | DL | DL |
|  | 8 | 8.7 | 33.6 | DL | DL | DL | DL | DL | DL | DL | DL | DL | DL | DL | 0.062 | DL | DL |
|  | 14 | 8.6 | 43 | DL | DL | DL | DL | DL | DL | DL | 0.001 | DL | DL | DL | 0.115 | DL | DL |
|  | 28 | 8.6 | 63.6 | DL | DL | DL | DL | 0.064 | DL | DL | 0.002 | DL | DL | DL | 0.198 | DL | DL |
|  | 57 | 7.8 | 91 | DL | DL | DL | DL | 0.084 | DL | DL | 0.003 | DL | DL | DL | 0.282 | DL | DL |
|  | 73 | 6.7 | 85.7 | DL | DL | DL | DL | 0.076 | DL | DL | 0.003 | DL | DL | DL | 0.248 | DL | DL |

**SI Table S3.** Average phase composition determined by SEM-EDS spot analysis performed on the unreacted Ca_2_SiO_4_ phase within BOF slag particles and the Ca-Si-H phase that replaces Ca_2_SiO_4_ in the surface alteration zone**.**

| **Element** | **A.Ca_2_SiO_4_** | **B. Ca-Si-H** | **Enrichment factor** |
| --- | --- | --- | --- |
|  | n = 17 | n = 89 |  |
|  | **Mol % ± 1σ** | **Mol % ± 1σ** | **(B/A)** |
| O | 56.4 ±2.7 | 55.6 ±9.2 | 1.0 |
| Mg | 0.13 ±0.09 | 0.41 ±0.77 | 3.2 |
| Al | 0.20 ±0.14 | 0.70 ±0.33 | 3.4 |
| Si | 11.8 ±0.65 | 17.7 ±5.9 | 1.5 |
| P | 1.41 ±0.08 | 4.45 ±1.46 | 3.2 |
| S | n.d.* | 0.16 ±0.14 | - |
| Cl | n.d.* | 0.12 ±0.07 | - |
| Ca | 27.4 ±1.4 | 16.1 ±5.1 | 0.6 |
| Sc | 0.19 ±0.04 | 0.12 ±0.08 | 0.6 |
| Ti | 0.14 ±0.10 | 0.50 ±0.28 | 3.6 |
| V | 0.22 ±0.22 | 0.28 ±0.35 | 1.3 |
| Mn | 0.05 ±0.02 | 0.25 ±0.44 | 5.5 |
| Fe | 0.50 ±0.11 | 2.43 ±2.43 | 5.0 |
| W | 0.06 ±0.01 | 0.13±0.04 | 2.2 |
| *Total* | *98.5* | *99.0* |  |

*not detected.

**SI Table S4.** Chemical composition of phases in the surface alteration zone as a function of distance from the surface. Measured by SEM-EDS.

| Size Fraction | Phase | Distance from surface | O | Mg | Al | Si | P | S | Cl | Ca | Sc | Ti | V | Mn | Fe | Lu | W |
| --- | --- | --- | --- | --- | --- | --- | --- | --- | --- | --- | --- | --- | --- | --- | --- | --- | --- |
|  |  | (μm) | mol % | | | | | | | | | | | | | | |
| Sand (0.5-1.0 mm) | Ca-Si-H | 0 | 67.25 | 0.21 | 0.69 | 15.30 | 3.26 | 0.04 | 0.12 | 10.81 | 0.07 | 0.40 | 0.12 | 0.14 | 1.52 | -0.02 | 0.09 |
|  | Ca-Si-H | 3 | 49.11 | 0.24 | 0.78 | 24.01 | 3.88 | 0.06 | 0.12 | 17.92 | 0.12 | 0.74 | 0.23 | 0.15 | 2.43 | 0.04 | 0.18 |
|  | Ca-Si-H | 5 | 38.82 | 0.24 | 0.83 | 31.47 | 3.84 | ND | 0.20 | 19.61 | 0.12 | 1.04 | 0.24 | 0.17 | 3.21 | ND | 0.23 |
|  | Ca-Si-H | 10 | 49.61 | 0.33 | 0.72 | 26.09 | 2.62 | 0.18 | 0.17 | 15.13 | ND | 0.89 | 0.33 | 0.21 | 3.65 | ND | 0.07 |
|  | Ca-Si-H | 15 | 43.79 | 0.29 | 0.86 | 27.52 | 3.63 | 0.07 | 0.20 | 18.03 | 0.13 | 0.63 | 0.20 | 0.26 | 4.26 | -0.04 | 0.18 |
|  | Ca-Si-H | 18 | 59.47 | 0.36 | 0.65 | 21.80 | 3.52 | 0.07 | 0.12 | 11.70 | 0.08 | 0.38 | 0.10 | 0.10 | 1.53 | 0.00 | 0.12 |
|  | Ca-Si-H | 25 | 39.27 | 0.26 | 0.52 | 23.23 | 2.86 | ND | 0.17 | 14.63 | 0.10 | 0.59 | 0.23 | 0.14 | 2.20 | ND | 0.15 |
|  | Ca-Si-H | 29 | 55.93 | 0.41 | 0.56 | 24.75 | 3.93 | ND | 0.11 | 11.79 | 0.11 | 0.40 | 0.12 | 0.08 | 1.67 | ND | 0.14 |
|  | Ca-Si-H | 34 | 57.13 | 0.42 | 0.50 | 23.12 | 4.00 | ND | 0.11 | 12.35 | ND | 0.41 | 0.10 | 0.11 | 1.64 | ND | 0.13 |
|  | Ca-Si-H | 40 | 55.98 | 0.39 | 0.46 | 23.83 | 3.98 | ND | 0.15 | 12.86 | ND | 0.38 | 0.14 | 0.08 | 1.58 | ND | 0.16 |
|  | Ca-Si-H | 46 | 65.06 | 0.51 | 0.58 | 20.14 | 2.76 | 0.16 | 0.08 | 8.71 | 0.07 | 0.28 | 0.08 | 0.09 | 1.36 | 0.01 | 0.08 |
|  | Ca-Si-H/Ca_2_SiO_4_ | 52 | 62.32 | 0.19 | 0.19 | 11.32 | 1.39 | 0.06 | ND | 23.50 | 0.14 | 0.07 | 0.09 | 0.06 | 0.54 | ND | 0.05 |
|  | Ca_2_SiO_4_ | 60 | 57.38 | ND | 0.14 | 12.08 | 1.50 | ND | ND | 27.91 | 0.17 | 0.09 | 0.11 | 0.04 | 0.53 | ND | 0.05 |
|  | Ca_2_SiO_4_ | 63 | 52.07 | 0.23 | ND | 10.99 | 1.32 | ND | ND | 25.70 | 0.26 | 0.07 | 0.19 | 0.04 | 0.53 | 0.00 | 0.06 |
|  | Ca_2_SiO_4_ | 65 | 50.47 | ND | 0.16 | 10.66 | 1.30 | ND | ND | 24.82 | 0.24 | 0.07 | 0.16 | 0.06 | 0.54 | ND | ND |
|  | Ca-Si-H | 0 | 55.84 | 0.30 | 0.65 | 23.12 | 4.41 | ND | 0.22 | 12.74 | ND | 0.39 | 0.12 | 0.17 | 1.87 | 0.00 | 0.11 |
|  | Ca-Si-H | 3 | 44.99 | 0.35 | 0.55 | 26.44 | 4.82 | ND | 0.19 | 18.88 | 0.10 | 0.56 | 0.15 | 0.17 | 2.58 | ND | 0.23 |
|  | Ca-Si-H | 6 | 36.24 | 0.50 | 0.67 | 31.73 | 5.29 | ND | 0.22 | 19.66 | 0.16 | 0.61 | 0.15 | 0.43 | 4.15 | ND | 0.19 |
|  | Ca-Si-H | 10 | 37.81 | 1.26 | 0.62 | 28.67 | 4.53 | 0.19 | 0.21 | 15.63 | 0.10 | 0.51 | 0.13 | 1.26 | 8.79 | ND | 0.20 |
|  | Ca-Si-H | 16 | 47.30 | 0.47 | 0.69 | 30.12 | 3.65 | ND | 0.19 | 12.88 | 0.08 | 0.62 | 0.15 | 0.34 | 3.22 | ND | 0.16 |
|  | Ca-Si-H | 20 | 56.86 | 0.34 | 0.82 | 26.10 | 2.63 | 0.12 | 0.17 | 9.76 | 0.09 | 0.57 | 0.10 | 0.17 | 2.18 | 0.00 | 0.08 |

**SI Table S4.** Continued.

| Size Fraction | Phase | Distance from surface | O | Mg | Al | Si | P | S | Cl | Ca | Sc | Ti | V | Mn | Fe | Lu | W |
| --- | --- | --- | --- | --- | --- | --- | --- | --- | --- | --- | --- | --- | --- | --- | --- | --- | --- |
|  |  | (μm) | mol % | | | | | | | | | | | | | | |
| Sand (0.5-1.0 mm) | Ca-Si-H | 0 | 65.06 | 0.51 | 0.47 | 18.69 | 3.27 | 0.11 | 0.12 | 9.29 | 0.07 | 0.28 | 0.08 | 0.40 | 1.45 | ND | 0.10 |
|  | Ca-Si-H | 5 | 58.32 | 0.72 | 0.59 | 24.48 | 3.20 | ND | 0.15 | 9.61 | ND | 0.44 | 0.09 | 0.11 | 2.16 | ND | 0.13 |
|  | Ca-Si-H | 19 | 53.42 | 0.80 | 0.69 | 31.03 | 1.99 | ND | 0.17 | 8.50 | 0.56 | 0.07 | 0.04 | 0.10 | 2.55 | ND | 0.15 |
|  | Ca-Si-H | 37 | 42.48 | 0.40 | 0.38 | 22.43 | 5.57 | ND | 0.10 | 24.71 | 0.16 | 0.48 | 0.24 | 0.22 | 2.67 | ND | 0.16 |
|  | Ca-Si-H | 41 | 58.02 | 0.29 | 0.92 | 14.62 | 4.19 | 0.05 | 0.05 | 17.86 | 0.15 | 0.63 | 0.36 | 0.20 | 2.51 | ND | 0.11 |
|  | Ca-Si-H | 49 | 45.29 | 0.24 | 0.26 | 10.14 | 2.92 | ND | 0.06 | 11.26 | 0.08 | 0.18 | 0.10 | 0.06 | 1.07 | ND | 0.11 |
|  | Ca-Si-H | 52 | 44.59 | 0.35 | 0.81 | 16.15 | 5.75 | 0.07 | 0.10 | 26.08 | 0.15 | 0.56 | 0.55 | 0.31 | 4.26 | ND | 0.13 |
|  | Ca-Si-H | 61 | 52.57 | 0.49 | 0.40 | 22.62 | 5.18 | ND | 0.10 | 16.32 | 0.11 | 0.33 | 0.13 | 0.10 | 1.51 | ND | 0.13 |
|  | Ca-Si-H | 68 | 47.27 | 0.54 | 0.51 | 26.61 | 4.50 | 0.06 | 0.13 | 17.39 | 0.10 | 0.42 | 0.15 | 0.08 | 2.03 | ND | 0.17 |
|  | Ca-Si-H | 71 | 61.57 | 0.80 | 0.41 | 20.39 | 3.30 | 0.07 | 0.07 | 11.66 | 0.08 | 0.25 | 0.09 | 0.05 | 1.12 | ND | 0.11 |
|  | Ca-Si-H/Ca_2_SiO_4_ | 76 | 56.04 | 0.23 | 0.17 | 13.25 | 1.72 | ND | ND | 27.65 | 0.17 | 0.13 | 0.05 | 0.53 | ND | ND | 0.07 |
|  | Ca_2_SiO_4_ | 79 | 56.46 | ND | 0.13 | 12.34 | 1.51 | ND | ND | 28.62 | 0.19 | ND | 0.12 | 0.05 | 0.43 | ND | 0.07 |
|  | Ca_2_SiO_4_ | 82 | 57.33 | ND | 0.15 | 12.10 | 1.51 | ND | ND | 27.89 | 0.18 | 0.10 | 0.14 | 0.05 | 0.47 | ND | ND |
|  | Ca-Si-H | 0 | 61.93 | 0.44 | 0.53 | 14.90 | 4.24 | ND | 0.12 | 13.26 | ND | 0.24 | 0.12 | 0.57 | 3.49 | 0.02 | 0.10 |
|  | Ca-Si-H | 2 | 62.35 | 0.21 | 0.53 | 16.08 | 4.69 | ND | 0.06 | 14.11 | 0.11 | 0.24 | 0.13 | 0.13 | 1.26 | -0.02 | 0.12 |
|  | Ca-Si-H | 4 | 37.73 | 0.58 | 0.39 | 21.29 | 4.94 | ND | 0.06 | 19.54 | ND | 0.38 | 0.17 | 0.10 | 2.26 | 12.38 | 0.19 |
|  | Ca-Si-H | 7 | 26.63 | 0.55 | 0.39 | 22.48 | 5.34 | ND | 0.08 | 21.70 | ND | 0.34 | 0.26 | 3.23 | 18.66 | ND | 0.19 |
|  | Ca-Si-H | 11 | 37.51 | 0.19 | 0.39 | 17.89 | 4.75 | ND | 0.08 | 18.40 | 0.12 | 0.26 | 0.15 | 0.14 | 1.43 | -0.02 | 0.15 |
|  | Ca-Si-H | 16 | 42.89 | 0.24 | 0.35 | 23.89 | 5.84 | ND | 0.10 | 23.45 | 0.12 | 0.42 | 0.20 | 0.16 | 2.12 | ND | 0.22 |
|  | Ca-Si-H | 18 | 52.35 | 0.29 | 0.32 | 21.43 | 4.89 | ND | 0.06 | 17.99 | 0.08 | 0.32 | 0.19 | 0.17 | 1.74 | ND | 0.16 |
|  | Ca-Si-H | 22 | 55.20 | 0.36 | 0.38 | 22.93 | 4.39 | ND | 0.09 | 14.32 | 0.07 | 0.33 | 0.12 | 0.10 | 1.57 | ND | 0.14 |
|  | Ca-Si-H | 28 | 61.15 | 0.40 | 0.44 | 22.21 | 3.42 | ND | 0.12 | 10.32 | 0.08 | 0.28 | 0.09 | 0.08 | 1.31 | ND | 0.11 |
|  | Ca-Si-H | 33 | 53.74 | 0.25 | 0.30 | 18.19 | 2.67 | ND | 0.06 | 22.59 | 0.15 | 0.29 | 0.13 | 0.10 | 1.34 | ND | 0.14 |
|  | Ca-Si-H/Ca_2_SiO_4_ | 36 | 58.90 | 0.23 | 0.23 | 15.47 | 2.22 | ND | 0.03 | 21.60 | 0.12 | 0.14 | 0.11 | 0.06 | 0.80 | ND | 0.10 |
|  | Ca_2_SiO_4_ | 38 | 58.29 | 0.06 | 0.10 | 12.00 | 1.44 | ND | ND | 27.24 | 0.15 | 0.07 | 0.08 | 0.05 | 0.46 | ND | 0.06 |
|  | Ca_2_SiO_4_ | 44 | 57.28 | ND | 0.11 | 12.23 | 1.48 | ND | ND | 28.12 | 0.19 | ND | 0.10 | 0.04 | 0.39 | ND | 0.07 |

**SI Table S4.** Continued.

| Size Fraction | Phase | Distance from surface | O | Mg | Al | Si | P | S | Cl | Ca | Sc | Ti | V | Mn | Fe | Lu | W |
| --- | --- | --- | --- | --- | --- | --- | --- | --- | --- | --- | --- | --- | --- | --- | --- | --- | --- |
|  |  | (μm) | mol % | | | | | | | | | | | | | | |
| Gravel (2.0-5.0 mm) | Ca-Si-H | 0 | 48.10 | 0.51 | 0.71 | 21.90 | 4.68 | 0.06 | 0.16 | 20.14 | 0.14 | 0.83 | 0.19 | 0.14 | 2.29 | ND | 0.16 |
|  | Ca-Si-H | 3 | 52.03 | 0.45 | 0.54 | 19.30 | 3.76 | ND | 0.14 | 20.09 | 0.16 | 0.88 | 0.17 | 0.07 | 2.28 | ND | 0.15 |
|  | Ca-Si-H | 5 | 41.22 | 0.55 | 0.48 | 23.08 | 4.60 | 0.09 | 0.20 | 25.00 | 0.17 | 1.06 | 0.21 | 0.10 | 3.05 | ND | 0.19 |
|  | Ca-Si-H | 3 | 59.69 | 0.45 | 0.56 | 17.39 | 4.05 | 0.05 | 0.15 | 15.12 | ND | 0.41 | 0.12 | 0.08 | 1.81 | ND | 0.10 |
|  | Ca-Si-H | 2 | 67.08 | 0.08 | 1.50 | 15.79 | 3.49 | ND | 0.15 | 8.21 | 0.06 | 1.01 | 0.12 | 0.13 | 2.24 | ND | 0.07 |
|  | Ca-Si-H | 3 | 58.48 | 0.18 | 1.36 | 13.86 | 5.21 | 0.24 | 0.12 | 13.96 | 0.09 | 0.67 | 0.27 | 0.24 | 5.14 | ND | 0.09 |
|  | Ca-Si-H | 2 | 51.91 | 0.37 | 0.92 | 10.38 | 2.90 | 0.04 | 0.08 | 7.02 | 0.05 | 0.41 | 0.05 | 0.17 | 1.82 | ND | 0.07 |
|  | Ca-Si-H | 8 | 55.01 | 0.20 | 0.97 | 13.19 | 7.15 | 0.07 | 0.10 | 19.60 | 0.15 | 0.56 | 0.15 | 0.16 | 2.41 | ND | 0.15 |
|  | Ca-Si-H | 12 | 61.21 | 0.14 | 0.67 | 8.82 | 6.46 | 0.08 | 0.07 | 18.21 | 0.11 | 0.35 | 0.17 | 0.29 | 3.04 | ND | 0.09 |
|  | Ca-Si-H | 17 | 56.68 | 0.17 | 0.76 | 10.69 | 7.74 | 0.24 | 0.07 | 20.40 | 0.11 | 0.38 | 0.15 | 0.23 | 2.09 | ND | 0.13 |
|  | Ca-Si-H | 23 | 63.36 | 0.20 | 0.48 | 7.60 | 6.16 | 0.31 | 0.06 | 19.20 | 0.11 | 0.30 | 0.12 | 0.19 | 1.75 | ND | 0.09 |
|  | Ca-Si-H | 30 | 68.69 | 0.17 | 0.58 | 8.25 | 6.44 | 0.27 | 0.04 | 13.69 | 0.08 | 0.22 | 0.08 | 0.11 | 1.04 | ND | 0.09 |
|  | Ca-Si-H | 39 | 58.98 | 0.14 | 0.62 | 10.96 | 8.03 | 0.16 | 0.09 | 18.32 | 0.14 | 0.36 | 0.11 | 0.17 | 1.57 | ND | 0.14 |
|  | Ca-Si-H | 48 | 63.14 | 0.18 | 1.72 | 19.36 | 3.19 | 0.11 | 0.14 | 8.94 | ND | 0.75 | 0.13 | 0.16 | 2.06 | ND | 0.08 |
|  | Ca-Si-H | 63 | 59.38 | 0.28 | 0.35 | 17.68 | 3.89 | 0.13 | 0.03 | 16.11 | 0.09 | 0.31 | 0.15 | 0.10 | 1.28 | ND | 0.12 |
|  | Ca-Si-H | 66 | 69.45 | 0.36 | 0.38 | 14.47 | 3.95 | 0.11 | 0.02 | 10.07 | 0.07 | 0.16 | 0.08 | 0.05 | 0.66 | ND | 0.10 |
|  | Ca-Si-H | 69 | 62.32 | 0.22 | 0.33 | 18.34 | 4.16 | 0.11 | ND | 13.01 | 0.08 | 0.18 | 0.11 | 0.07 | 0.86 | ND | 0.12 |
|  | Ca-Si-H | 72 | 66.14 | 0.24 | 0.36 | 18.80 | 2.81 | ND | ND | 10.45 | 0.07 | 0.15 | 0.09 | 0.05 | 0.65 | ND | 0.10 |
|  | Ca-Si-H/Ca_2_SiO_4_ | 75 | 71.34 | 0.07 | ND | 10.63 | 1.31 | ND | ND | 15.88 | 0.12 | 0.07 | 0.03 | 0.34 | ND | ND | 0.04 |
|  | Ca_2_SiO_4_ | 78 | 60.35 | ND | 0.19 | 11.95 | 1.35 | ND | ND | 25.41 | 0.17 | ND | 0.10 | 0.03 | 0.39 | ND | 0.05 |
|  | Ca_2_SiO_4_ | 83 | 59.63 | ND | 0.17 | 12.00 | 1.34 | ND | ND | 26.01 | 0.18 | ND | 0.10 | 0.04 | 0.40 | ND | 0.06 |
|  | Ca_2_SiO_4_ | 90 | 57.99 | 0.09 | 0.20 | 12.06 | 1.47 | ND | ND | 26.64 | 0.31 | ND | 0.13 | 0.10 | 0.77 | ND | 0.05 |

**SI Table S4.** Continued.

| Size Fraction | Phase | Distance from surface | O | Mg | Al | Si | P | S | Cl | Ca | Sc | Ti | V | Mn | Fe | Lu | W |
| --- | --- | --- | --- | --- | --- | --- | --- | --- | --- | --- | --- | --- | --- | --- | --- | --- | --- |
|  |  | (μm) | mol % | | | | | | | | | | | | | | |
| Gravel (2.0-5.0 mm) | Ca-Si-H | 0 | 73.76 | 0.16 | 1.49 | 13.59 | 1.77 | 0.06 | 0.51 | 6.55 | 0.05 | 0.40 | 0.06 | 0.09 | 1.52 | ND | ND |
|  | Ca-Si-H | 1.5 | 65.11 | 0.17 | 1.71 | 18.65 | 2.69 | 0.06 | 0.16 | 8.55 | 0.53 | ND | 0.07 | 0.11 | 2.11 | ND | 0.08 |
|  | Ca-Si-H | 9 | 61.02 | 0.20 | 0.89 | 13.36 | 6.78 | 0.14 | 0.11 | 14.76 | 0.12 | 0.41 | 0.12 | 0.17 | 1.82 | ND | 0.12 |
|  | Ca-Si-H | 19 | 57.59 | 0.15 | 0.85 | 15.37 | 6.37 | 0.08 | 0.07 | 16.15 | 0.12 | 0.56 | 0.11 | 0.15 | 2.27 | ND | 0.16 |
|  | Ca-Si-H | 28 | 61.18 | 0.12 | 0.61 | 11.91 | 7.34 | ND | 0.13 | 16.18 | 0.10 | 0.36 | 0.12 | 0.13 | 1.60 | ND | 0.13 |
|  | Ca-Si-H | 38 | 58.24 | 0.09 | 0.44 | 9.96 | 8.76 | ND | 0.11 | 20.02 | 0.15 | 0.30 | 0.15 | 0.18 | 1.50 | ND | 0.08 |
|  | Ca-Si-H | 56 | 58.36 | 0.19 | 0.40 | 9.75 | 8.50 | 0.38 | 0.05 | 19.96 | 0.13 | 0.26 | 0.15 | 0.17 | 1.38 | ND | 0.13 |
|  | Ca-Si-H | 69 | 64.58 | 0.36 | 0.75 | 13.30 | 4.83 | 0.32 | 0.08 | 12.37 | ND | 0.32 | 0.12 | 0.24 | 2.21 | ND | 0.10 |
|  | Ca-Si-H | 69 | 48.90 | 7.30 | 0.71 | 11.70 | 3.53 | 0.22 | 0.08 | 10.11 | ND | 0.22 | 0.08 | 2.38 | 14.04 | ND | 0.08 |
|  | Ca-Si-H | 88 | 61.38 | 0.28 | 0.50 | 15.86 | 5.72 | 0.54 | 0.12 | 13.06 | 0.10 | 0.29 | 0.10 | 0.14 | 1.45 | ND | 0.10 |
|  | Ca-Si-H | 106 | 65.82 | 0.28 | 0.37 | 12.77 | 5.28 | 0.58 | 0.13 | 12.74 | 0.07 | 0.24 | 0.10 | 0.12 | 1.13 | ND | 0.11 |
|  | Ca-Si-H | 125 | 56.46 | 0.34 | 0.42 | 17.26 | 5.76 | 0.45 | 0.07 | 16.75 | 0.12 | 0.27 | 0.13 | 0.12 | 1.35 | ND | 0.15 |
|  | Ca-Si-H | 144 | 57.79 | 0.38 | 0.39 | 17.90 | 5.40 | 0.32 | 0.04 | 15.45 | 0.11 | 0.28 | 0.14 | 0.08 | 1.22 | ND | 0.15 |
|  | Ca-Si-H/Ca_2_SiO_4_ | 156 | 50.21 | ND | 0.11 | 10.05 | 1.34 | ND | ND | 22.77 | 0.13 | 0.08 | 0.09 | 0.05 | 0.43 | ND | ND |
|  | Ca_2_SiO_4_ | 163 | 57.31 | ND | 0.17 | 11.82 | 1.45 | ND | ND | 28.11 | 0.17 | 0.10 | 0.16 | 0.04 | 0.56 | ND | 0.06 |
|  | Ca_2_SiO_5_ | 181 | 57.22 | ND | 0.10 | 12.30 | 1.46 | ND | ND | 28.14 | 0.20 | ND | 0.10 | 0.04 | 0.40 | ND | 0.05 |
|  | Ca_2_SiO_6_ | 206 | 57.16 | ND | 0.11 | 12.35 | 1.41 | ND | ND | 28.25 | 0.18 | ND | 0.11 | 0.02 | 0.37 | ND | 0.05 |

**SI Table S4.** Continued.

| Size Fraction | Phase | Distance from surface | O | Mg | Al | Si | P | S | Cl | Ca | Sc | Ti | V | Mn | Fe | Lu | W |
| --- | --- | --- | --- | --- | --- | --- | --- | --- | --- | --- | --- | --- | --- | --- | --- | --- | --- |
|  |  | (μm) | mol % | | | | | | | | | | | | | | |
| Blocks (20 x 10 x 10 mm) | Ca-Si-H | 0 | 70.66 | 0.13 | 1.47 | 11.50 | 3.73 | 0.06 | 0.07 | 9.73 | 0.05 | 0.73 | 0.10 | 0.08 | 1.57 | ND | 0.06 |
|  | Ca-Si-H | 0 | 71.39 | 0.13 | 1.11 | 8.10 | 4.80 | 0.13 | 0.20 | 11.18 | ND | 0.65 | 0.30 | 0.19 | 1.74 | ND | 0.09 |
|  | Ca-Si-H | 0 | 53.78 | 0.30 | 1.55 | 14.90 | 7.53 | ND | 0.19 | 18.02 | ND | 0.92 | 0.21 | 0.21 | 2.22 | ND | 0.12 |
|  | Ca-Si-H | 1 | 56.62 | 0.39 | 0.72 | 14.84 | 4.74 | 0.11 | 0.17 | 19.38 | ND | 0.56 | 0.50 | 0.08 | 1.75 | ND | 0.13 |
|  | Ca-Si-H | 2 | 63.87 | 0.46 | 0.56 | 13.48 | 4.37 | ND | 0.13 | 15.09 | 0.10 | 0.34 | 0.29 | 0.05 | 1.18 | ND | 0.07 |
|  | Ca-Si-H | 2 | 51.53 | 0.71 | 0.97 | 13.99 | 4.39 | 0.53 | 0.28 | 21.52 | 0.14 | 0.72 | 0.65 | 0.41 | 4.01 | ND | 0.11 |
|  | Ca-Si-H | 3 | 51.84 | 0.30 | 0.59 | 14.07 | 4.69 | 0.06 | 0.12 | 24.77 | 0.15 | 0.70 | 0.68 | 0.04 | 1.81 | ND | 0.17 |
|  | Ca-Si-H | 8 | 56.87 | 0.33 | 0.62 | 14.09 | 4.80 | ND | 0.10 | 20.36 | 0.15 | 0.52 | 0.54 | 0.07 | 1.43 | ND | 0.13 |
|  | Ca-Si-H | 15 | 54.30 | 0.24 | 0.87 | 15.19 | 4.47 | ND | 0.08 | 21.50 | 0.14 | 0.74 | 0.78 | 0.08 | 1.49 | ND | 0.12 |
|  | Ca-Si-H | 24 | 42.35 | 0.16 | 1.41 | 16.55 | 3.41 | 0.12 | 0.08 | 28.32 | 0.15 | 1.62 | 1.48 | 0.24 | 3.98 | ND | 0.14 |
|  | Ca-Si-H | 33 | 55.85 | 0.18 | 1.07 | 13.61 | 3.53 | 0.33 | 0.05 | 19.71 | 0.14 | 0.88 | 1.29 | 0.35 | 2.92 | ND | 0.10 |
|  | Ca-Si-H | 42 | 50.52 | 0.24 | 0.86 | 16.16 | 3.89 | ND | 0.05 | 22.11 | ND | 1.04 | 1.30 | 0.55 | 3.13 | ND | 0.15 |
|  | Ca-Si-H | 51 | 59.57 | ND | 0.46 | 11.84 | 1.64 | ND | ND | 24.50 | 0.16 | 0.41 | 0.69 | 0.05 | 0.62 | ND | 0.06 |
|  | Ca-Si-H | 57 | 49.35 | ND | 0.93 | 13.02 | 3.27 | 0.04 | ND | 28.62 | 0.16 | 0.87 | 1.85 | 0.08 | 1.74 | ND | 0.09 |
|  | Ca-Si-H/Ca_2_SiO_4_ | 60 | 56.90 | ND | 0.77 | 11.31 | 1.75 | 0.05 | ND | 26.85 | 0.15 | 0.39 | 0.86 | 0.05 | 0.86 | ND | 0.06 |
|  | Ca_2_SiO_4_ | 63 | 51.03 | ND | 0.57 | 10.17 | 1.31 | ND | ND | 26.39 | 0.14 | 0.26 | 0.77 | 0.04 | 0.46 | ND | 0.07 |
|  | Ca_2_SiO_5_ | 72 | 55.65 | ND | 0.54 | 11.10 | 1.40 | ND | ND | 29.20 | 0.16 | 0.37 | 0.76 | 0.05 | 0.72 | ND | 0.06 |
|  | Ca_2_SiO_6_ | 78 | 56.15 | ND | 0.19 | 12.12 | 1.37 | ND | ND | 29.00 | 0.17 | 0.15 | 0.31 | 0.04 | 0.46 | ND | 0.05 |

**SI Table S4.** Continued.

| Size Fraction | Phase | Distance from surface | O | Mg | Al | Si | P | S | Cl | Ca | Sc | Ti | V | Mn | Fe | Lu | W |
| --- | --- | --- | --- | --- | --- | --- | --- | --- | --- | --- | --- | --- | --- | --- | --- | --- | --- |
|  |  | (μm) | mol % | | | | | | | | | | | | | | |
| Blocks (20 x 10 x 10 mm) | Ca-Si-H | 0 | 65.12 | 0.31 | 0.62 | 11.67 | 3.68 | ND | 0.29 | 13.43 | ND | 0.31 | 0.28 | 1.20 | 2.96 | ND | 0.11 |
|  | Ca-Si-H | 1 | 72.27 | 0.34 | 0.50 | 10.21 | 3.49 | ND | 0.10 | 11.39 | ND | 0.28 | 0.20 | 0.12 | 0.93 | ND | 0.07 |
|  | Ca-Si-H | 4 | 56.29 | 0.29 | 1.12 | 14.85 | 4.17 | 0.15 | 0.15 | 18.64 | 0.10 | 0.60 | 0.54 | 0.72 | 2.31 | ND | 0.09 |
|  | Ca-Si-H | 9 | 63.47 | 0.25 | 0.63 | 13.16 | 4.16 | ND | 0.13 | 15.44 | 0.11 | 0.46 | 0.41 | 0.27 | 1.39 | ND | 0.11 |
|  | Ca-Si-H | 18 | 60.92 | 0.18 | 0.59 | 13.08 | 4.30 | ND | 0.14 | 18.01 | 0.10 | 0.56 | 0.59 | 0.11 | 1.32 | ND | 0.10 |
|  | Ca-Si-H | 22 | 56.35 | 0.19 | 0.94 | 16.01 | 3.70 | ND | 0.05 | 18.89 | 0.11 | 1.00 | 0.98 | 0.13 | 1.51 | ND | 0.13 |
|  | Ca-Si-H | 25 | 51.85 | 0.21 | 1.05 | 16.49 | 3.64 | 0.05 | 0.05 | 21.36 | 0.14 | 1.22 | 1.46 | 0.20 | 2.13 | ND | 0.11 |
|  | Ca-Si-H/Ca_2_SiO_4_ | 28 | 58.13 | ND | 0.58 | 11.57 | 1.52 | ND | 0.03 | 25.99 | ND | 0.51 | 0.79 | 0.06 | 0.75 | ND | 0.05 |
|  | Ca_2_SiO_4_ | 32 | 56.37 | ND | 0.23 | 12.19 | 1.26 | ND | ND | 28.84 | 0.17 | 0.13 | 0.26 | 0.04 | 0.43 | ND | 0.05 |


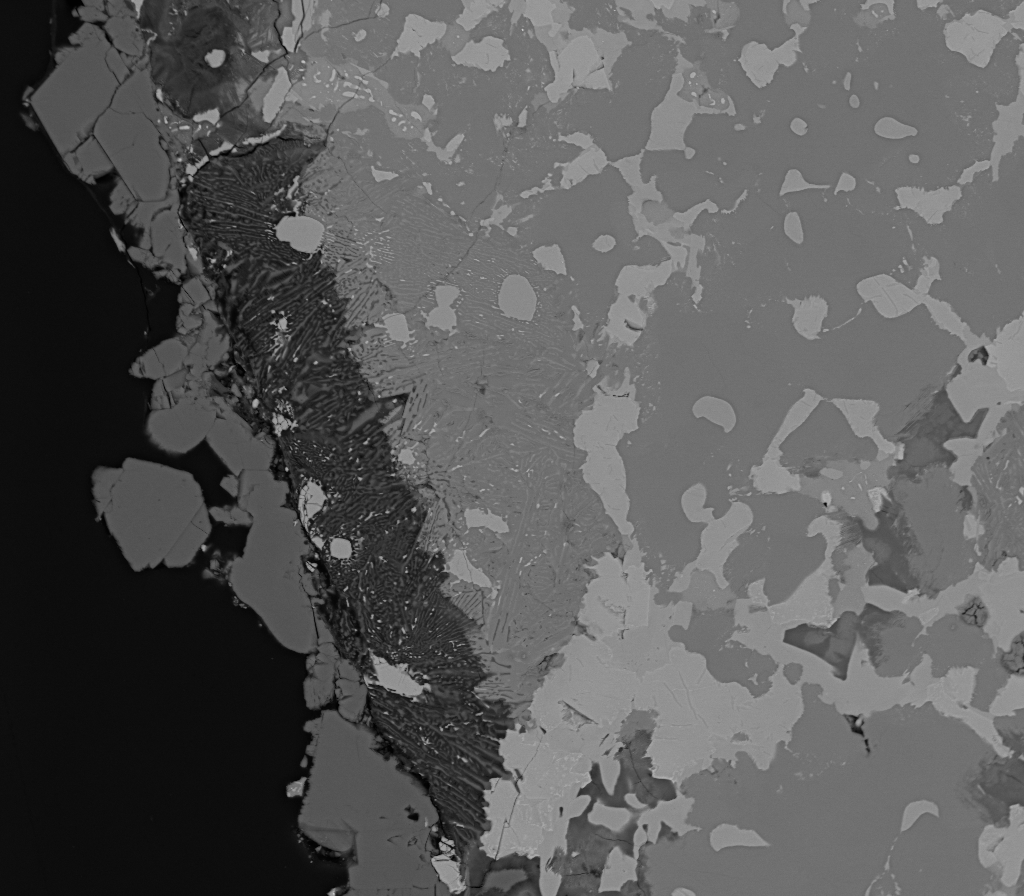


B

L

Alteration
 rind

V

W

Ca_2_Si

100 µm

Unweathered Slag

CaCO_3_

CaSiH

**SI Figure S1.** Example BSEI electron micrograph showing the primary (Ca_2_S – larnite; B – Brownmillerite; L – Lime; W – Wusite; V – void space) and secondary Ca-Si-H and CaCO_3_ phases present at the surface of the aerobically weathered 20 mm BOF slag blocks after 6 months total immersion. All phases where identified by EDS spot analysis of representative regions. Alteration depths were defined as the changed surface region within the original volume of the slag particle (presence of refractory phases allows good estimation of the original particle size); the thickness of any CaCO_3_ layer was not included in the analysis.


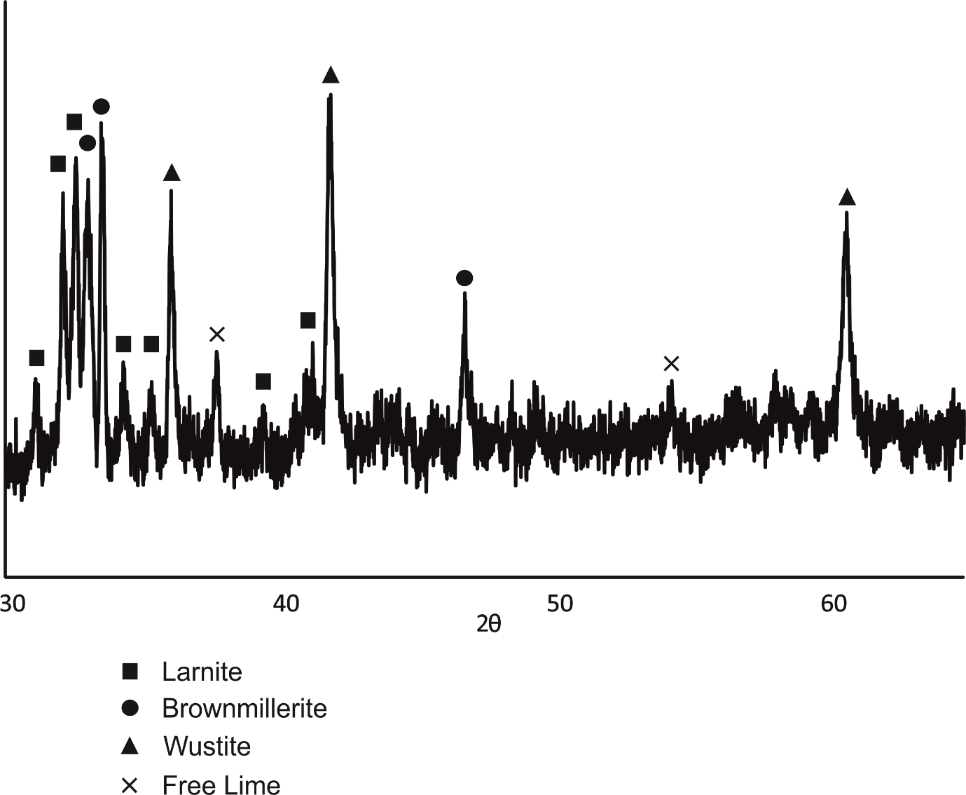


**SI Figure S2.** XRD pattern collected from the crushed steel slag sample annotated with major phase peaks detected.

| 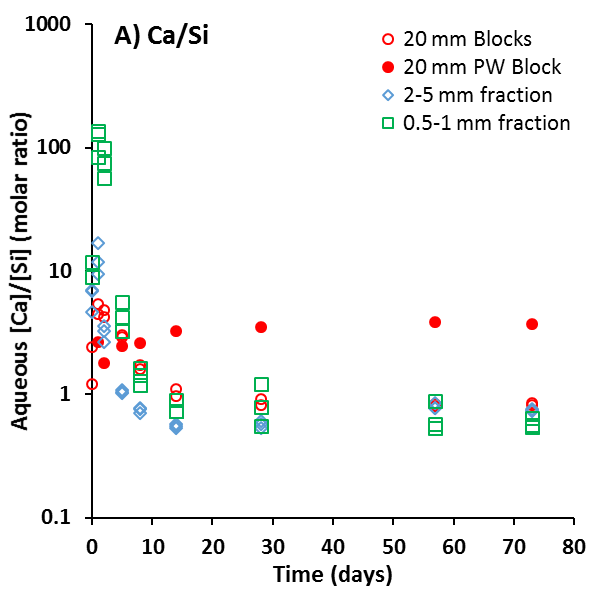 | 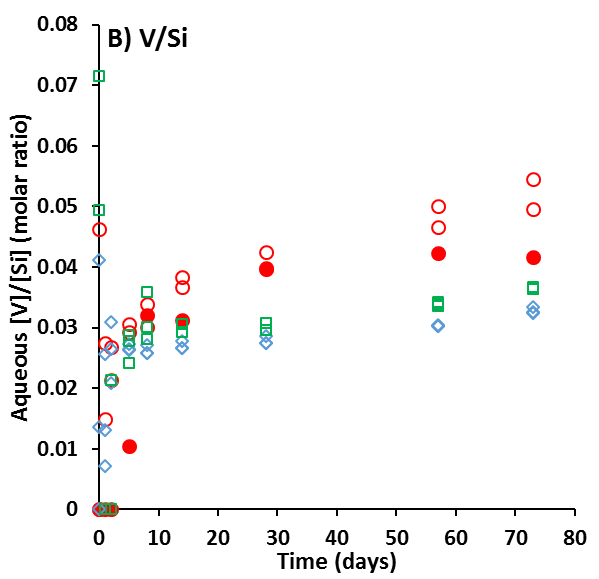 |
| --- | --- |

**SI Figure S3.** Elemental cross-plots showing; A) The relationships between aqueous [Ca] and [Si], and; B) Aqueous [Si] and [V] in individual replicates during the leaching tests.


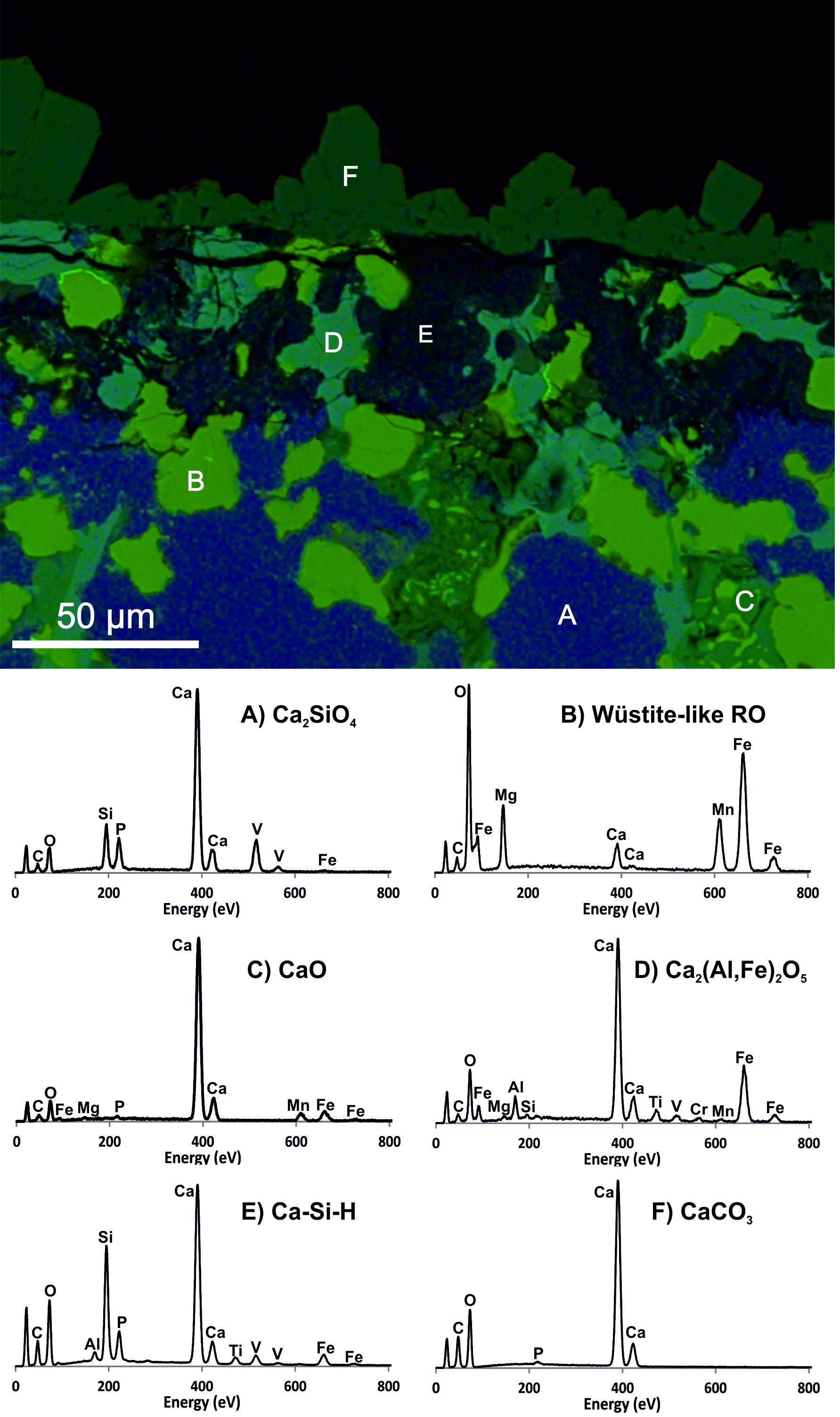


**SI Figure S4.** Composite false colour SEM-EDS elemental map showing phase discrimination within the 6 month pre-weathered BOF slag block. A-D) Example EDS spectra collected from each of the 6 major phases detected with the slag; and E-F) Example EDS spectra from neo-formed phases present in the altered surface layer.

**
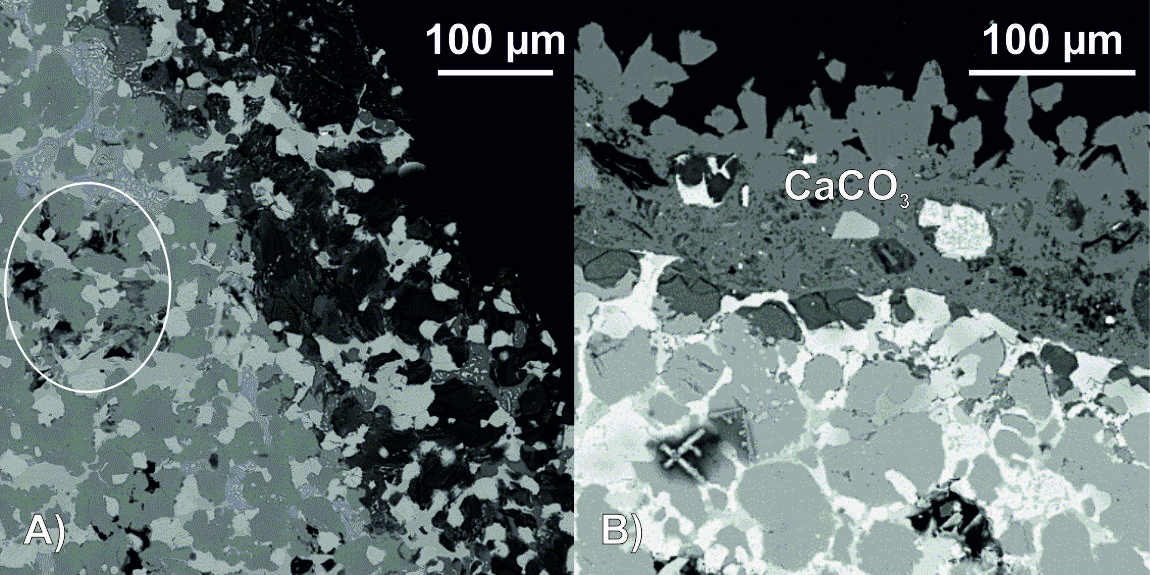
**

**SI Figure S5.** BSE images of different sized BOF slag particles after leaching for 73 days; (a) Block showing possible Ca-Si-H formation within occasional voids remote from the block surface, and (b) Sand-sized fraction showing CaCO_3_ crystals on the weathered surface.
